# Pretreatment MRI and Ultrasound Features of the Primary Tumor and Axillary Nodes for Predicting Axillary Pathologic Complete Response After Neoadjuvant Chemotherapy: A Cross-Anatomic Comparison

**DOI:** 10.3390/diagnostics16172853

**Published:** 2026-09-04

**Authors:** Shuang Liu, Yongxin Chen, Wenjie Tang, Yonghui Feng, Liwen Pan, Jiaxin Chen, Jingxuan Guo, Weifeng Liu, Qingcong Kong, Xinqing Jiang

**Affiliations:** 1 Department of Radiology, School of Medicine, The Second Affiliated Hospital (Guangzhou First People’s Hospital), South China University of Technology, No. 1 Panfu Road, Guangzhou 510180, China; 202420157123@mail.scut.edu.cn (S.L.); yongxinchen@stu2023.jnu.edu.cn (Y.C.); eywenjietang@scut.edu.cn (W.T.); plwdoctor@163.com (L.P.); 202520156193@mail.scut.edu.cn (J.C.); 2 Department of Radiology, Guangzhou Panyu Maternal and Child Health Hospital, Guangzhou 511483, China; 13611421228@163.com; 3 Clinical Medical College, Tianjin Medical University, Tianjin 300270, China; 13821912036@163.com; 4 Department of Radiology, The Third Affiliated Hospital, Sun Yat-Sen University, Guangzhou 510630, China

**Keywords:** breast cancer, magnetic resonance imaging, axillary pathologic complete response, ultrasound, neoadjuvant chemotherapy

## Abstract

**Background/Objectives:** Pretreatment imaging may help estimate axillary response after neoadjuvant chemotherapy (NAC) in clinically node-positive (cN+) breast cancer. This study compared routinely available magnetic resonance imaging (MRI) and ultrasound (US) descriptors from the primary tumor and axillary nodes for predicting axillary nodal response, defined as postneoadjuvant pathologic node-negative status (ypN0). **Methods:** This retrospective multicenter study included 243 patients from three centers: 146 in the training cohort, 59 in the internal validation cohort, and 38 in the external validation cohort. The index breast tumor and axillary node were identified by a senior radiologist, and two radiologists independently assessed structured descriptors based on BI-RADS 2025. Least absolute shrinkage and selection operator regression and logistic regression were used to develop imaging-only, clinical-only, and clinicoradiologic models. Performance was evaluated using area under the curve (AUC), calibration, decision curve analysis, net reclassification improvement, and integrated discrimination improvement. **Results:** In the internal and external validation cohorts, the trimodal imaging model achieved AUCs of 0.761 and 0.806, and the clinicoradiologic model achieved the highest AUCs of 0.858 and 0.931, respectively. In pooled validation subgroup analyses, AUCs were 0.928, 0.804, and 0.700 for HR-positive/HER2-negative, HER2-positive, and triple-negative tumors, and 0.898 and 0.880 for cN1 and cN2–3 disease, respectively. **Conclusions:** MRI tumor descriptors provided the strongest imaging signal for ypN0 prediction, while US descriptors offered modest complementary information. The clinicoradiologic model may support pretreatment risk stratification and multidisciplinary planning for post-NAC axillary reassessment, but should not independently determine sentinel lymph node biopsy, axillary lymph node dissection, or omission of axillary staging.

## 1. Introduction

For patients with clinically node-positive (cN+) breast cancer, neoadjuvant chemotherapy (NAC) followed by surgery is widely used in current clinical practice [1]. In addition to reducing the size of the primary breast tumor, NAC can eradicate metastatic disease in axillary lymph nodes (ALNs) and thereby create opportunities for breast-conserving surgery and axillary downstaging [2]. However, the likelihood of nodal eradication after NAC is heterogeneous. Molecular subtype is an established determinant of achieving ypN0, defined as no residual nodal metastasis after NAC, but subtype alone does not fully capture patient-level response [3,4]. Posttreatment axillary status is also closely related to long-term disease-free survival and may be more informative prognostically than nodal status at initial presentation [4,5]. Therefore, identifying patients who are likely to achieve ypN0 before surgery using pretreatment clinical and imaging information is clinically relevant, especially in the context of ongoing efforts to reduce the extent of axillary surgery.

Axillary lymph node dissection (ALND) remains the reference standard for axillary restaging after NAC [6]. However, ALND is associated with well-recognized morbidity, including lymphedema and upper-extremity dysfunction [7,8]. Moreover, because pathologic confirmation is obtained only at surgery, ALND cannot provide pretreatment or preoperative information for individualized planning. Sentinel lymph node biopsy (SLNB) has been investigated as a less invasive approach in selected patients [9]. However, the SENTINA and ACOSOG Z1071 trials reported false-negative rates of 14.2% and 12.6%, respectively, for post-NAC SLNB in cN+ disease, both exceeding the commonly accepted threshold of 10% [6,10]. Targeted axillary dissection (TAD), which combines SLNB with selective removal of the pretreatment-marked node, has been shown to reduce the false-negative rate for axillary restaging in initially cN+ patients who convert to ycN0 after NAC [11]. These limitations and developments support the need for reliable noninvasive tools that can estimate the likelihood of ypN0 before surgery and assist individualized axillary management.

Dynamic contrast-enhanced magnetic resonance imaging (DCE-MRI) is among the most sensitive imaging modalities for assessing treatment response in the primary breast tumor. It offers excellent soft-tissue contrast with multiplanar coverage of the primary tumor and surrounding tissues, facilitating standardized assessment of morphologic and signal-intensity descriptors [12,13]. In routine breast MRI, however, axillary evaluation can be constrained, as the axilla may be incompletely covered or nodal morphology may be insufficiently depicted in a subset of patients, particularly without dedicated axillary coils or standardized axillary protocols [14]. Ultrasound (US), by contrast, offers high spatial resolution for direct visualization of cortical changes and the hilum, and is therefore commonly used as a first-line tool for morphologic assessment of ALNs [15,16]. Descriptor-based US assessment is inherently reader-dependent, underscoring the importance of standardized definitions and reporting conventions. In routine practice, one-to-one matching between individual suspicious nodes and postoperative specimens is often infeasible when multiple nodes are involved; therefore, an imaging-defined “index” node is commonly used as a pragmatic surrogate for axillary status [17]. Taken together, these modality-specific characteristics suggest a practical complementarity: MRI excels at comprehensive characterization of the primary tumor, whereas US is well suited for targeted morphologic assessment of ALNs within routine workflows.

A growing body of work has explored integrating MRI assessment of the primary tumor with ultrasound (US) evaluation of axillary lymph nodes to predict axillary response after neoadjuvant chemotherapy, leveraging the complementary strengths of cross-modality and cross-anatomic information [17,18,19]. A recent multimodal nomogram also combined clinical and imaging features to predict axillary pathologic complete response in a comparable cN+ population [20]. However, that study focused on an overall nomogram that incorporated posttreatment response-related variables. Because breast and axillary US are typically performed in the same clinical encounter [21], the operational question extends beyond any single pairing (e.g., “MRI tumor + US axilla”): among routinely available, workflow-neutral strategies based on structured descriptors, which anatomic–modality combinations are most informative for pretreatment ypN0 prediction? Key evidence gaps remain. First, within the same cN+ population, systematic head-to-head comparisons of pretreatment descriptor models from MRI tumor, US tumor, and US axillary nodal assessments—and their alternative fusion strategies—are limited, leaving the optimal integration pathway unclear. Second, incremental benefit is often incompletely characterized; few studies quantify the added value of incorporating US tumor and/or nodal descriptors beyond the best unimodal model under a consistent evaluation framework, including reclassification- and discrimination-oriented metrics.

Against this background, we conducted a head-to-head evaluation of pretreatment structured descriptor models to predict ypN0 after NAC in cN+ breast cancer. Specifically, within an “anatomic region × imaging modality” framework, we compared unimodal models derived from MRI tumor, US tumor, and US axillary nodal descriptors with their alternative combinations, and quantified incremental value beyond the best unimodal strategy using discrimination- and reclassification-oriented metrics. We further integrated the optimal imaging strategy with selected clinicopathologic variables to construct a joint model for preoperative assessment. This design allowed direct comparison of routinely reported anatomic–modality descriptor modules within a multicenter cohort that included independent external validation.

## 2. Materials and Methods

### 2.1. Study Population

This retrospective multicenter study was approved by the Institutional Review Boards of Guangzhou First People’s Hospital (Approval No. S2023-083-01), The Third Affiliated Hospital of Sun Yat-sen University (Approval No. RG2024-070-01), and Guangzhou Panyu Maternal and Child Health Hospital (Approval No. 2026090113). The requirement for written informed consent was waived owing to the retrospective nature of the study. Consecutive patients with nonmetastatic early-stage breast cancer who underwent NAC followed by surgery between July 2017 and August 2026 were screened from three centers. Treatment regimens followed the National Comprehensive Cancer Network guidelines [22]. Detailed neoadjuvant systemic therapy regimens according to molecular subtype are provided in Supplementary Methods and Supplementary Table S16.

**Figure 1:**
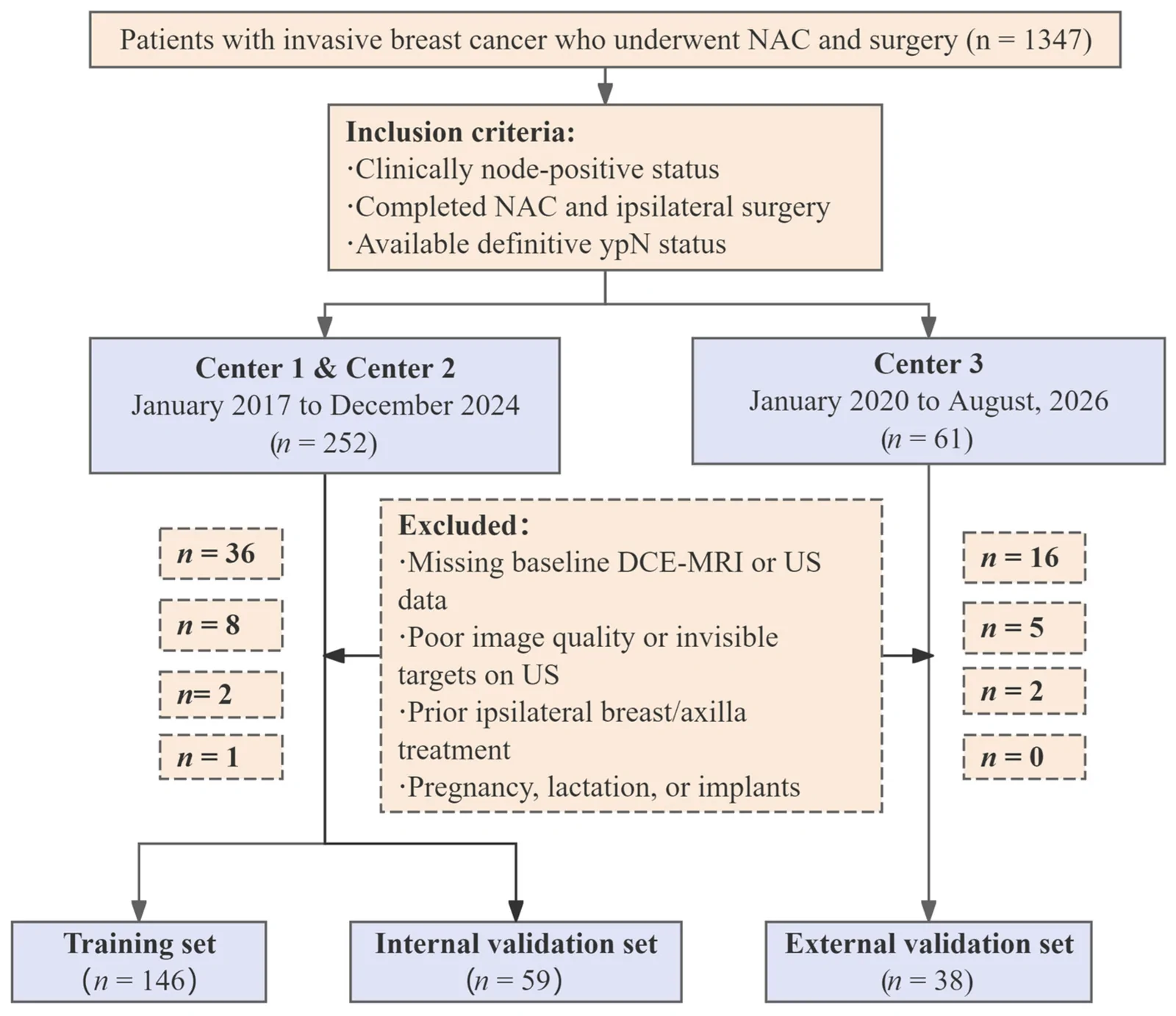
Flowchart of patient inclusion and cohort allocation. NAC, neoadjuvant chemotherapy; ypN status, postneoadjuvant pathologic nodal status; DCE-MRI, dynamic contrast-enhanced magnetic resonance imaging; US, ultrasound.

Inclusion criteria were: (a) histopathologically confirmed primary invasive breast cancer; (b) clinical evaluation (needle biopsy/conventional imaging/palpation) indicating cN+; (c) completion of NAC followed by ipsilateral breast and axillary surgery; and (d) definitive postoperative pathology for ypN0 determination. Exclusion criteria were: (a) absence of baseline DCE-MRI or US; (b) inadequate image quality or nonvisualization of measurable primary tumor or ALNs on US; (c) prior ipsilateral breast/axillary treatment; or (d) pregnancy, lactation, or breast implants. Patients from Centers 1 and 2 were randomly assigned to the training cohort (*n* = 146) and internal validation cohort (*n* = 59) at a 7:3 ratio. Patients from Center 3 were used as an external validation cohort (*n* = 38). Finally, 243 patients were included in the analysis. Patient enrollment and cohort allocation are summarized in Figure 1.

### 2.2. Image Acquisition

**Figure 2:**
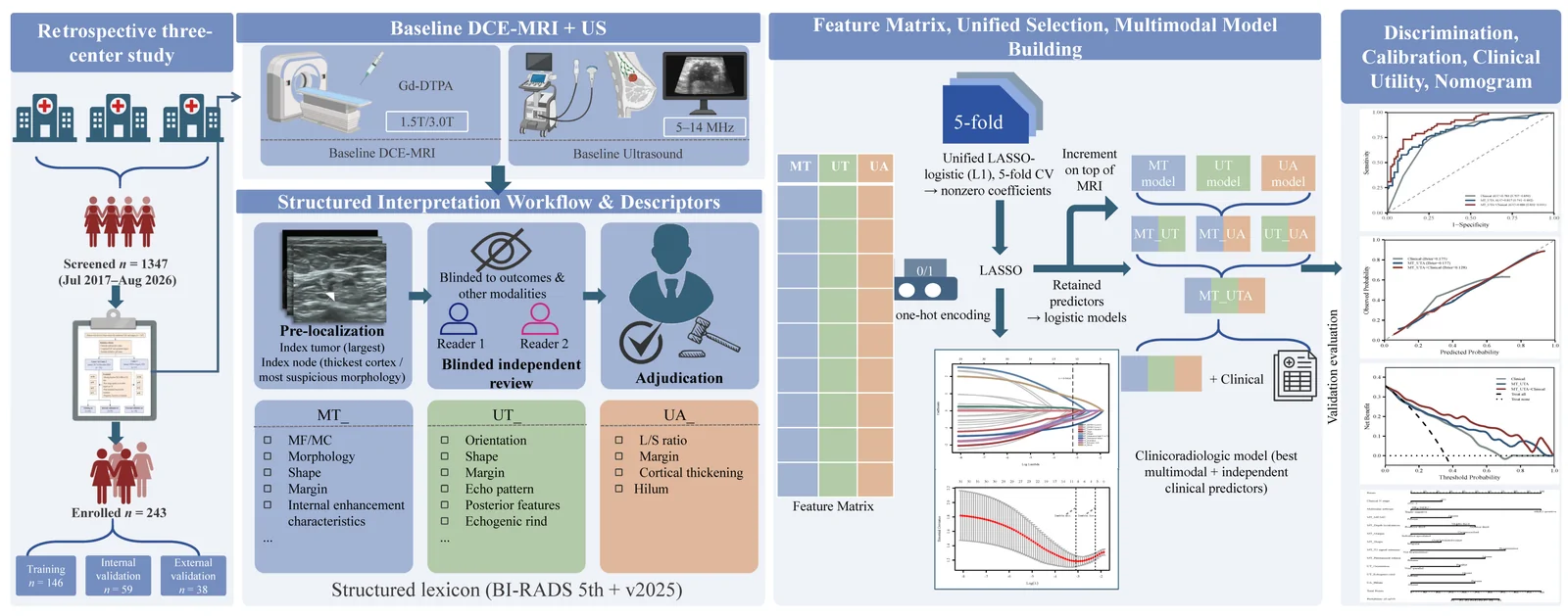
Overview of the study workflow. Baseline DCE-MRI and ultrasound descriptors were grouped as MRI tumor (MT), US tumor (UT), and US axilla (UA), and entered into a unified LASSO logistic regression framework to build unimodal, bimodal, and trimodal models, with additional clinical integration. Performance was evaluated by discrimination, calibration, and clinical utility. DCE-MRI, dynamic contrast-enhanced magnetic resonance imaging; LASSO, least absolute shrinkage and selection operator.

The workflow of this study is depicted in Figure 2. US examinations were performed using Hitachi ultrasound systems (Hitachi Medical Corporation, Tokyo, Japan) or Resona 9 systems (Shenzhen Mindray Bio-Medical Electronics Co., Ltd., Shenzhen, China) (5–14 MHz linear transducers). Breast MRI was performed on 1.5-T scanners (uMR 560, Shanghai United Imaging Healthcare Co., Ltd., Shanghai, China; Achieva, Philips Medical Systems Nederland B.V., Best, The Netherlands; MAGNETOM Aera, Siemens Healthineers, Erlangen, Germany) or a 3.0-T scanner (Discovery MR750, GE Healthcare, Waukesha, WI, USA). DCE-MRI was acquired after axial unenhanced fat-suppressed T1-weighted imaging. Gd-DTPA (Magnevist, Bayer Pharma AG, Berlin, Germany) was administered as a bolus (0.2 mL/kg; 1.5 mL/s) followed by a 20-mL saline flush. Detailed acquisition parameters are provided in Supplementary Table S1.

### 2.3. Image Interpretation and Analysis

A senior breast radiologist (15 years’ experience) developed a study-specific structured descriptor set based on the American College of Radiology Breast Imaging Reporting and Data System lexicon (5th edition and 2025 Manual) and relevant literature [23,24,25,26]. Two breast radiologists (3 and 5 years’ experience) completed standardized training and calibration on 20 independent cases before formal review.

Image interpretation followed a pre-localization and independent blinded review workflow. Using de-identified baseline images only and blinded to outcomes and postoperative pathology, the senior radiologist pre-identified the index tumor (largest invasive lesion) and index node (most suspicious ALN based on cortical thickening, hilum abnormality, shape, and margin). In multifocal/multicentric disease or when multiple suspicious nodes were present, corresponding targets were marked to align MRI and US assessment of the same lesions. The two readers then independently assessed the pre-specified targets on MRI, breast US, and axillary US, blinded to clinical information, pathology, and other-modality images; disagreements were resolved by the senior radiologist under the same blinded conditions.

MRI descriptors included lesion type, shape, margin, internal enhancement, T2 signal intensity, peritumoral edema, enhancement kinetics, depth localization, multifocal/multicentric disease, fibroglandular tissue, and background parenchymal enhancement. Breast US descriptors included orientation, shape, margin, echo pattern, posterior features, echogenic rind, and calcifications. Axillary US descriptors included long-to-short axis ratio, margin, cortical thickening, and hilum status. Detailed classification criteria and operational definitions are provided in Supplementary Tables S2–S4 [26,27,28].

In the multimodality analyses, MRI tumor, US tumor, and US axillary descriptors were denoted as MT, UT, and UA, respectively.

### 2.4. Clinical and Pathologic Data Collection

Clinical and pathologic data were retrospectively obtained from the electronic medical record and pathology systems. Clinical variables included age, menopausal status, baseline clinical T stage, baseline clinical N stage, and neoadjuvant systemic therapy regimen. Pathologic variables included histology, grade, lymphovascular invasion, estrogen receptor (ER), progesterone receptor (PR), human epidermal growth factor receptor 2 (HER2), Ki-67 index, and molecular subtype, ypT stage, ypN stage, and ypCR status. Definitions are provided in Supplementary Table S5 [29,30,31,32,33,34,35]. The primary outcome was ypN0, with no residual micrometastases or macrometastases in resected ALNs.

### 2.5. Feature Selection and Model Development

The binary outcome was ypN0 versus ypN1–3. Feature selection and model construction were confined to the training cohort, with the internal and external validation cohorts used only for performance evaluation. To allow modality-level comparison within a common modeling framework, baseline DCE-MRI tumor, US tumor, and US axillary descriptors were entered together into a LASSO-penalized logistic regression model. Multi-level categorical variables were one-hot encoded. The regularization parameter λ was selected by 5-fold cross-validation, and predictors with nonzero coefficients were retained. These predictors were subsequently allocated to their respective modality groups and used to construct unimodal models for MRI tumor (MT), US tumor (UT), and US axilla (UA), bimodal models combining two groups (MT_UT, MT_UA, and UT_UA), and the trimodal model incorporating all three groups (MT_UTA). This strategy was used to ensure that modality-specific and multimodal models were compared within the same selected descriptor space, rather than being influenced by separate feature-selection procedures for each modality.

### 2.6. Clinical and Clinicoradiologic Models

Under the same outcome definition, candidate clinicopathologic variables were first assessed using univariable logistic regression in the training cohort. Variables with *p* < 0.05 were considered for multivariable logistic regression, with final inclusion determined by clinical relevance, the number of ypN0 events, and collinearity. When molecular subtype and ER, PR, and/or HER2 status were simultaneously significant, molecular subtype was retained because it incorporates these biomarkers. The resulting predictors were used to construct the clinical-only model (Clinical). The clinicoradiologic model (MT_UTA + Clinical) was then developed by integrating these independent clinical predictors with the imaging predictors selected from the best-performing multimodal model. To evaluate its incremental value, MT_UTA + Clinical was compared with the imaging-only model (MT_UTA) and the Clinical model. Finally, a nomogram was constructed from the regression coefficients to estimate the individualized probability of ypN0.

### 2.7. Statistical Analysis

Interobserver agreement for categorical imaging descriptors was assessed using Cohen’s κ (95% CI), with κ ≥ 0.75 indicating good agreement. Baseline characteristics were compared across the three cohorts using one-way ANOVA or the Kruskal–Wallis test for continuous variables and the χ^2^ test or Fisher’s exact test for categorical variables, as appropriate; two-group comparisons (e.g., by postneoadjuvant nodal status) were performed using the *t* test or Wilcoxon rank-sum test for continuous variables and the χ^2^ test or Fisher’s exact test for categorical variables. Univariable and multivariable logistic regression were used to assess associations between clinicopathologic variables and ypN0. Model performance was evaluated by AUC (95% CI); the training-cohort Youden cutoff was applied to the internal and external validation cohorts to calculate sensitivity, specificity, PPV, NPV, and accuracy. Incremental value was assessed by AUC differences, continuous NRI, and IDI. Calibration and clinical utility were evaluated using 1000-bootstrap calibration curves, the Brier score, and decision curve analysis. Associations between LASSO-selected imaging descriptors and clinicopathologic variables were evaluated using the χ^2^ test or Fisher’s exact test, with Cramer’s V used as the effect-size measure and FDR adjustment applied for multiple comparisons. Analyses were performed using R software (version 4.4.1; R Foundation for Statistical Computing, Vienna, Austria), with two-sided *p* < 0.05.

## 3. Results

### 3.1. Clinical and Pathologic Characteristics

A total of 243 cN+ breast cancer patients were included (mean age ± standard deviation, 51.84 ± 10.67), comprising 146 in the training cohort, 59 in the internal validation cohort, and 38 in the external validation cohort. The overall ypN0 rate was 39.09% (95/243). The cohort included 114 HR+/HER2− tumors (46.91%), 102 HER2-positive tumors (41.98%), and 27 triple-negative tumors (11.11%). Baseline clinical and pathologic characteristics are presented in Table 1. Most baseline characteristics were comparable across the three cohorts, except for clinical T stage (*p* = 0.014). The ypN0 rate did not differ significantly across cohorts (*p* = 0.350). Neoadjuvant systemic therapy regimens and postneoadjuvant pathologic characteristics are summarized in Supplementary Table S6.

### 3.2. Mri and Us Characteristics

The inter-reader agreement for the assessed MRI and US imaging features was good to excellent, with κ values ranging from 0.756 to 0.900 (Supplementary Table S7).

In the training cohort, on MRI, MF/MC disease was present in 47.26% (69/146) of patients, and most lesions were located in the middle third of the breast (54.11%, 79/146). Most MRI lesions presented as a mass (89.73%, 131/146), with heterogeneous or extremely dense fibroglandular tissue in 70.55% (103/146) and minimal or mild background parenchymal enhancement in 74.66% (109/146). For lesion morphology and enhancement, irregular shape, indistinct/spiculated margin, heterogeneous internal enhancement, and persistent/plateau enhancement kinetics were observed in 84.93% (124/146), 80.82% (118/146), 85.62% (125/146), and 75.34% (110/146) of lesions, respectively. Regarding T2-related findings, T2 hyperintensity was uncommon (8.90%, 13/146), whereas peritumoral edema was observed in 66.44% (97/146).

On breast US, tumor orientation, shape, and margin were predominantly parallel (82.19%, 120/146), irregular (76.03%, 111/146), and indistinct/angular/microlobulated/spiculated (93.84%, 137/146), respectively. For echotexture-related findings, hypoechoic and heterogeneous/mixed solid and cystic echo patterns accounted for 56.16% (82/146) and 43.84% (64/146), respectively. Among posterior and interface features, posterior shadowing was the most frequent posterior feature (63.70%, 93/146), while echogenic rind was observed in 53.42% (78/146) of tumors. Calcifications in a mass were present in 81.51% (119/146) of tumors. On axillary US, a long-to-short axis ratio less than 2, non-circumscribed nodal margin, non-uniform cortical thickening, and absent nodal hilum were observed in 63.01% (92/146), 41.10% (60/146), 35.62% (52/146), and 73.97% (108/146) of cases, respectively.

Several pretreatment imaging descriptors differed according to postneoadjuvant nodal status. Among MRI descriptors, patients with residual nodal disease (ypN1–3) more frequently showed indistinct/spiculated margin (87.23% vs. 69.23%, *p* = 0.008) and peritumoral edema (77.66% vs. 46.15%, *p* < 0.001) than those who achieved ypN0. Among US tumor descriptors, non-parallel orientation (23.40% vs. 7.69%, *p* = 0.017) and echogenic rind (63.83% vs. 34.62%, *p* < 0.001) were more frequent in the ypN1–3 group. For axillary US, absent nodal hilum was also more frequent in the ypN1–3 group (84.04% vs. 55.77%, *p* < 0.001) (Table 2). The distributions of candidate imaging descriptors between the training and internal validation cohorts and of LASSO-selected descriptors across all three cohorts are provided in Supplementary Tables S8 and S9.

### 3.3. Factors Associated with ypN0

In the training cohort, univariable analyses showed that cN stage, molecular subtype, ER status, PR status, and HER2 status were significantly associated with ypN0 (all *p* < 0.05); in multivariable analysis, cN stage and molecular subtype remained independent predictors (Table 3). Specifically, cN2–3 disease was associated with a lower likelihood of ypN0 than cN1 disease (OR = 0.44, 95% CI: 0.20–0.96; *p* = 0.040). Molecular subtype was also independently associated with ypN0 (overall *p* < 0.001); HER2-positive tumors showed a higher likelihood of ypN0 than HR+/HER2− tumors (OR = 9.03, 95% CI: 3.90–20.90; *p* < 0.001), whereas triple-negative tumors did not differ significantly from HR+/HER2− tumors (OR = 1.72, 95% CI: 0.46–6.37; *p* = 0.417).

**Figure 3:**
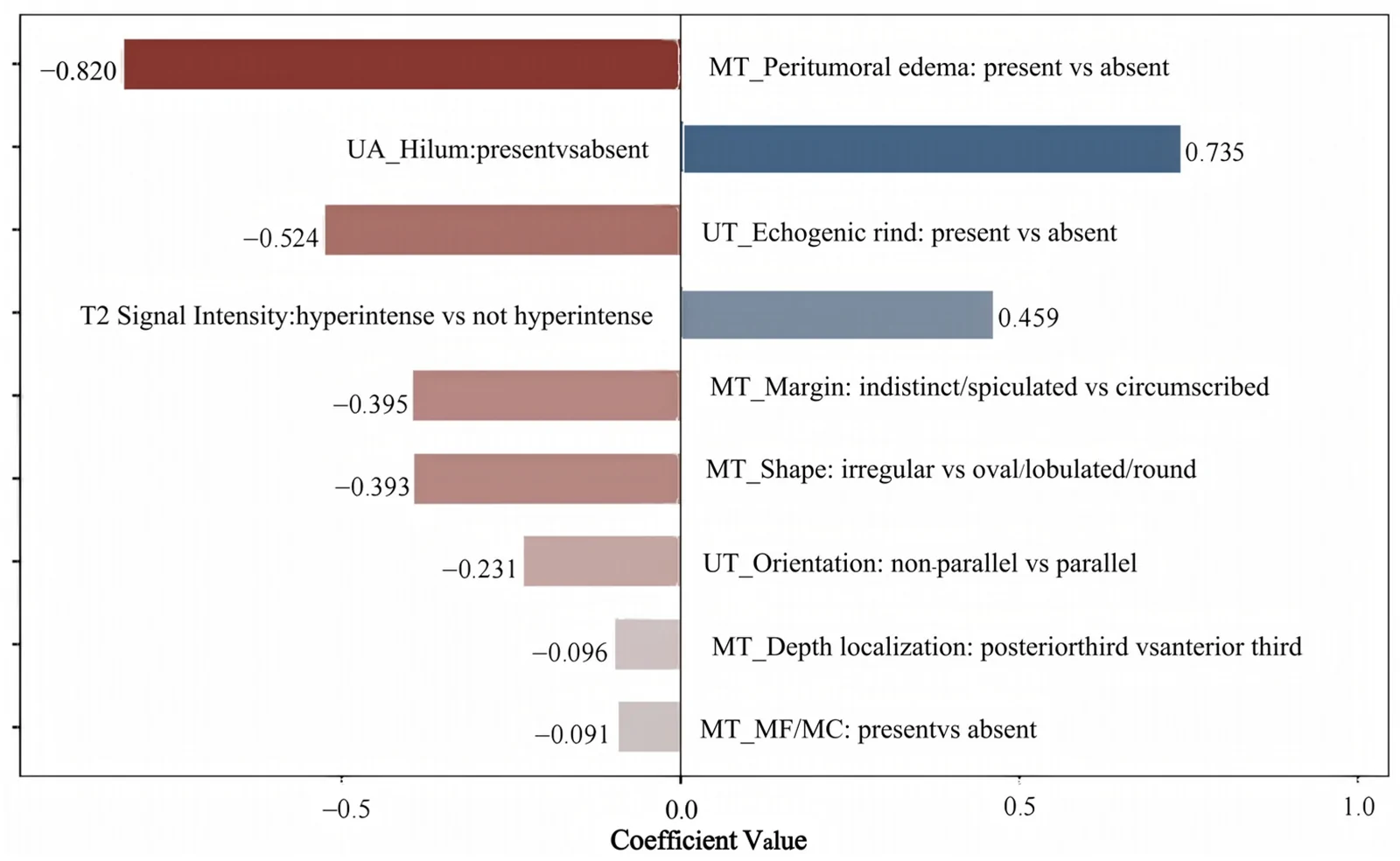
LASSO-selected feature coefficients for predicting ypN0. Variables are ranked by the absolute value of their coefficients. Positive coefficients indicate higher likelihood of ypN0, and negative coefficients indicate lower likelihood. Coefficients for categorical variables represent the contrast shown on the y-axis labels. MT = MRI tumor; UT = ultrasound tumor; UA = ultrasound axilla; MF/MC = multifocal and/or multicentric cancer.

LASSO logistic regression was applied to all MRI and US features, with the optimal penalty determined by 5-fold cross-validation (Figures S1 and S2). Nine imaging predictors were retained (Figure 3). Negative coefficients (lower probability of ypN0) were observed for MT_Peritumoral edema (present vs. absent), UT_Echogenic rind (present vs. absent), MT_Margin (indistinct/spiculated vs. circumscribed), MT_Shape (irregular vs. oval/lobulated/round), UT_Orientation (non-parallel vs. parallel), MT_Depth localization (posterior third vs. anterior third), and MT_MF/MC (present vs. absent), whereas positive coefficients (higher probability of ypN0) were observed for UA_Hilum (present vs. absent) and MT_T2 signal intensity (hyperintense vs. not hyperintense). Detailed LASSO coefficients and directions are provided in Supplementary Table S10. Associations between the LASSO-selected imaging descriptors and clinicopathologic variables are provided in Supplementary Table S11 and Figure S3. Exploratory analyses for ypCR and ypT0/is are provided in Supplementary Table S15.

### 3.4. Performance of Imaging Models for Predicting ypN0

In the training cohort, the MT model achieved an AUC of 0.761, exceeding the UT model (AUC = 0.673) and the UA model (AUC = 0.641). The MT model showed a sensitivity of 0.827, whereas the UA model showed a specificity of 0.840. Combining MRI and US information improved discrimination: the MT_UT model, MT_UA model, and MT_UTA model achieved AUCs of 0.804, 0.794, and 0.817, respectively, all higher than any single-modality model; the UT_UA model achieved an AUC of 0.717.

**Figure 4:**
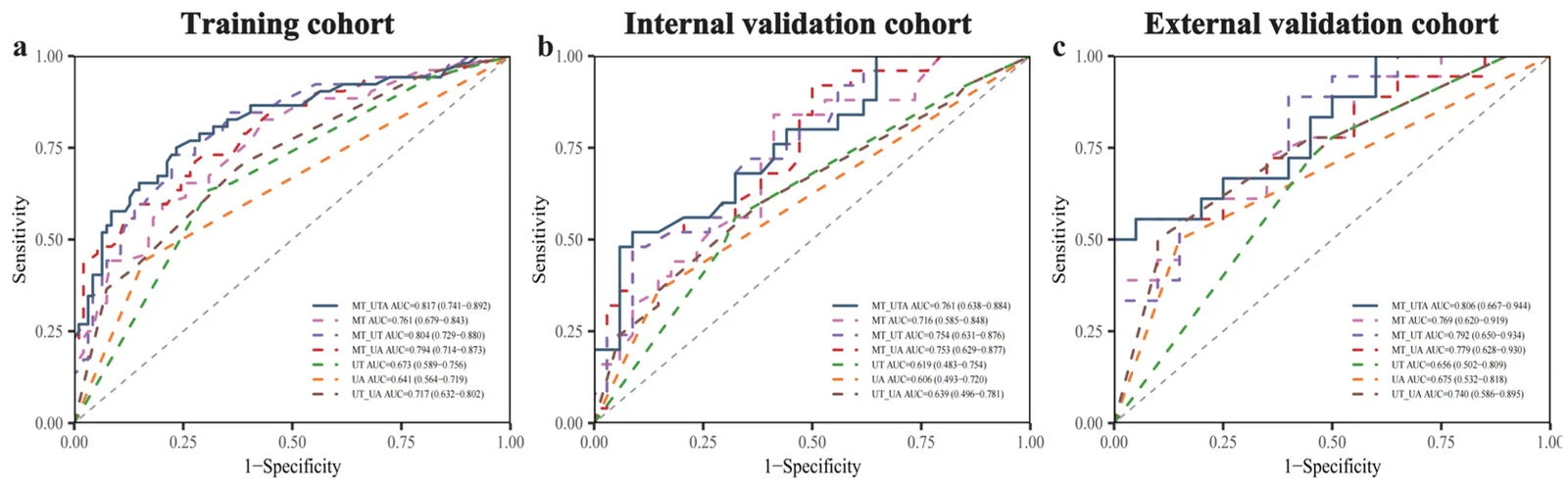
Receiver operating characteristic curves of the imaging models across cohorts. Curves are shown for the training (**a**), internal validation (**b**), and external validation (**c**) cohorts. AUC = area under the curve; MT = MRI tumor; UT = ultrasound tumor; UA = ultrasound axilla; MT_UT = MRI tumor + ultrasound tumor; MT_UA = MRI tumor + ultrasound axilla; UT_UA = ultrasound tumor + ultrasound axilla; MT_UTA = combination of all three feature sets.

In the internal validation cohort, the MT model achieved an AUC of 0.716, remaining higher than the UT model (AUC = 0.619) and the UA model (AUC = 0.606), while the MT_UT model, MT_UA model, and MT_UTA model achieved AUCs of 0.754, 0.753, and 0.761, respectively; for the MT_UTA model, specificity and sensitivity were 0.912 and 0.520. In the external validation cohort, MT again outperformed the UT and UA models (AUCs: 0.769, 0.656, and 0.675, respectively). The multimodal imaging models showed AUCs of 0.792 for MT_UT, 0.779 for MT_UA, 0.740 for UT_UA, and 0.806 for MT_UTA, with MT_UTA again showing the highest imaging-only performance. Detailed cutoffs and classification metrics are provided in Table 4 and Figure 4.

### 3.5. Incremental Value of Ultrasound Descriptors Beyond MRI Tumor

The MT model achieved an AUC of 0.761 in the training cohort and 0.716 in the internal validation cohort, and 0.769 in the external validation cohort. In the training cohort, incorporating US features resulted in incremental improvements in discrimination (Table 5). Compared with the MT model, the AUC increased by 0.044 for the MT_UT model (*p* = 0.07) and by 0.033 for the MT_UA model (*p* = 0.167), whereas the trimodal MT_UTA model achieved a larger AUC increase of 0.056, reaching statistical significance (*p* = 0.045). Reclassification metrics also improved, with NRI values of 0.631, 0.566, and 0.606 and IDI values of 0.059, 0.065, and 0.102 for the MT_UT, MT_UA, and MT_UTA models, respectively (all *p* ≤ 0.003).

In the internal and external validation cohorts, AUC, NRI, and IDI values for the MT_UT, MT_UA, and MT_UTA models were higher than those for the MT model, although the magnitude of improvement was modest (Table 5).

### 3.6. Performance of the Integrated Clinicoradiologic Model

**Figure 5:**
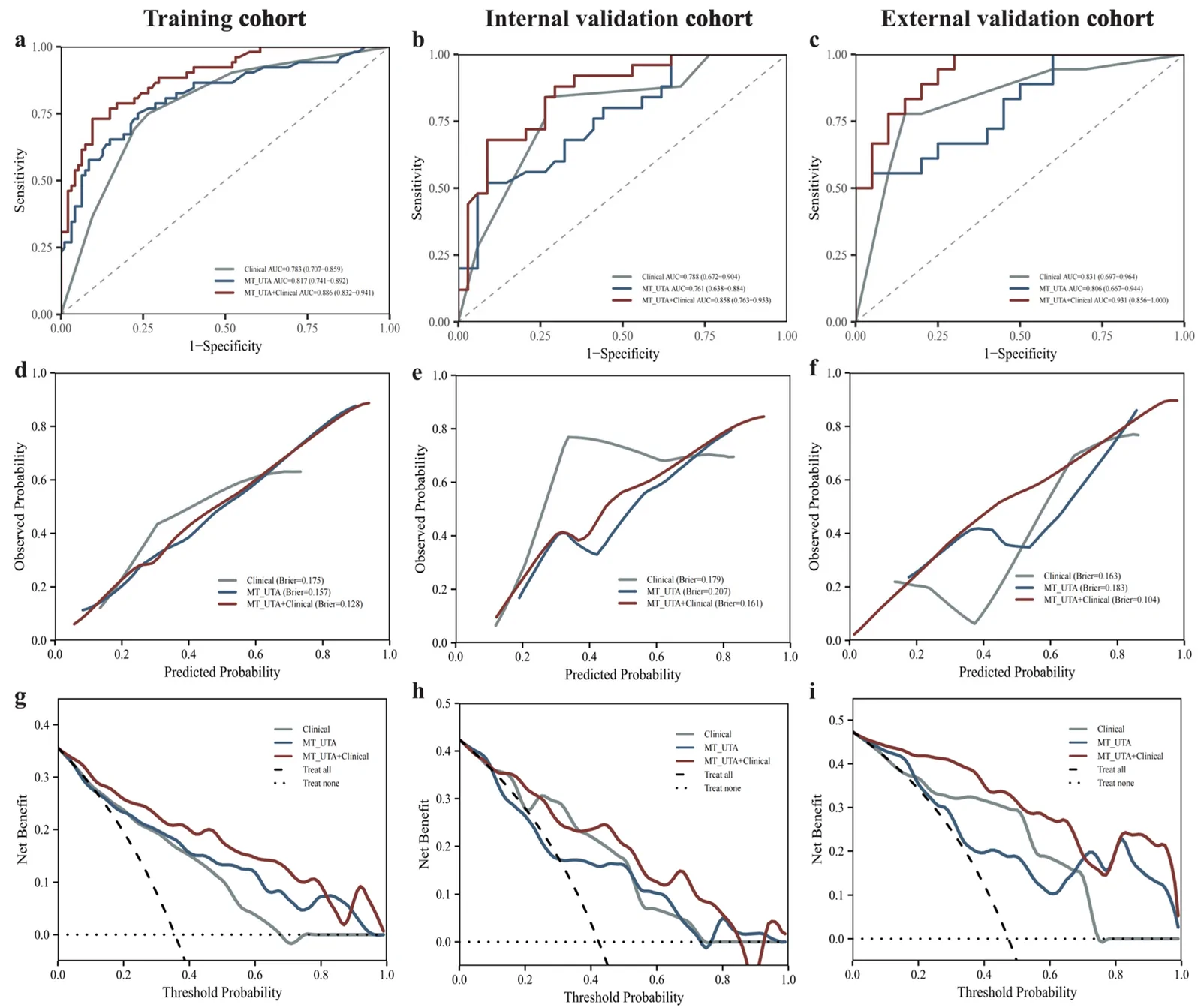
Performance of the clinical, imaging, and clinicoradiologic models for predicting ypN0. (**a**–**c**) Receiver operating characteristic curves in the training (**a**), internal validation (**b**), and external validation (**c**) cohorts; (**d**–**f**) calibration curves for the training (**d**), internal validation (**e**), and external validation (**f**) cohorts; and (**g**–**i**) decision curve analyses for the training (**g**), internal validation (**h**), and external validation (**i**) cohorts. Clinical = clinical-only model; MT_UTA = MRI tumor + ultrasound tumor + ultrasound axilla descriptors; MT_UTA + Clinical = clinicoradiologic model.

**Figure 6:**
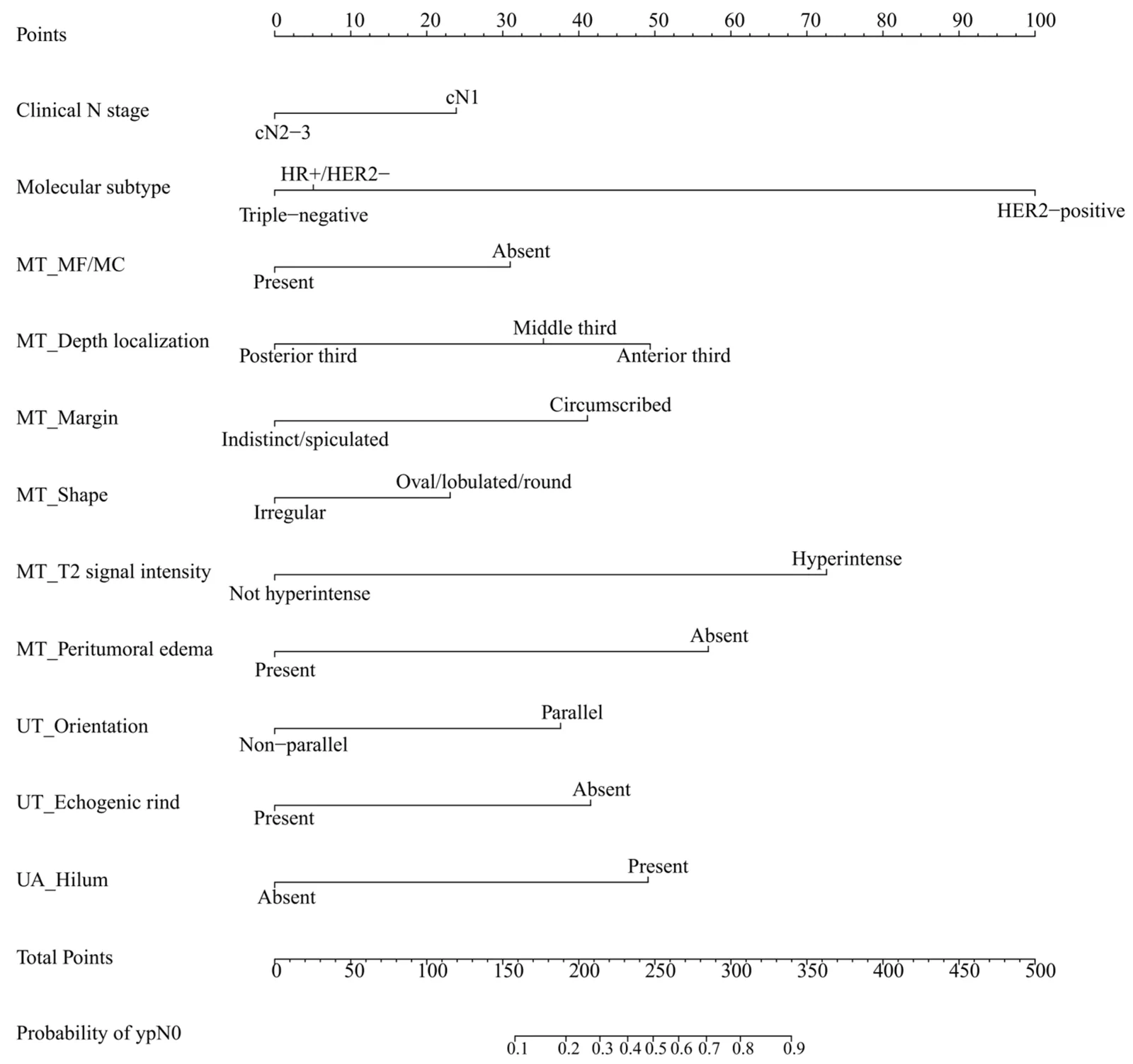
Nomogram for predicting ypN0 based on imaging and clinicopathologic variables. The training-cohort Youden cutoff of the MT_UTA + Clinical model corresponded to a predicted probability of 0.467, equivalent to approximately 243 total points on the nomogram; patients with total points at or above this threshold were classified as predicted ypN0, and those below as predicted ypN1–3. cN = clinical nodal stage; HER2 = human epidermal growth factor receptor 2; HR = hormone receptor; MF/MC = multifocal and/or multicentric disease; MT = MRI tumor; UA = US axilla; UT = US tumor.

The predictive performance of the Clinical model, the MT_UTA model, and the MT_UTA + Clinical model was evaluated (Table 4, Figure 5). Additional incremental analyses compared MT_UTA + Clinical with both the Clinical and MT_UTA models (Table 6). A nomogram based on the MT_UTA + Clinical model was constructed to estimate individualized probability of ypN0 (Figure 6). Full coefficient specifications of the Clinical and MT_UTA + Clinical models are provided in Supplementary Table S12.

In the training cohort, the AUCs were 0.783 for the Clinical model, 0.817 for the MT_UTA model, and 0.886 for the MT_UTA + Clinical model. In the internal validation cohort, the corresponding AUCs were 0.788, 0.761, and 0.858. In the external validation cohort, the corresponding AUCs were 0.831, 0.806, and 0.931. MT_UTA + Clinical achieved higher AUCs than the Clinical model across all three cohorts, with statistical significance reached in the training cohort. Significant AUC improvements were also observed over MT_UTA alone across all three cohorts (Table 6). Exploratory subgroup analyses in the pooled validation cohort showed AUCs of 0.928, 0.804, and 0.700 for HR+/HER2−, HER2-positive, and triple-negative tumors, respectively, and 0.898 and 0.880 for cN1 and cN2–3 disease, respectively (Supplementary Table S14).

**Figure 7:**
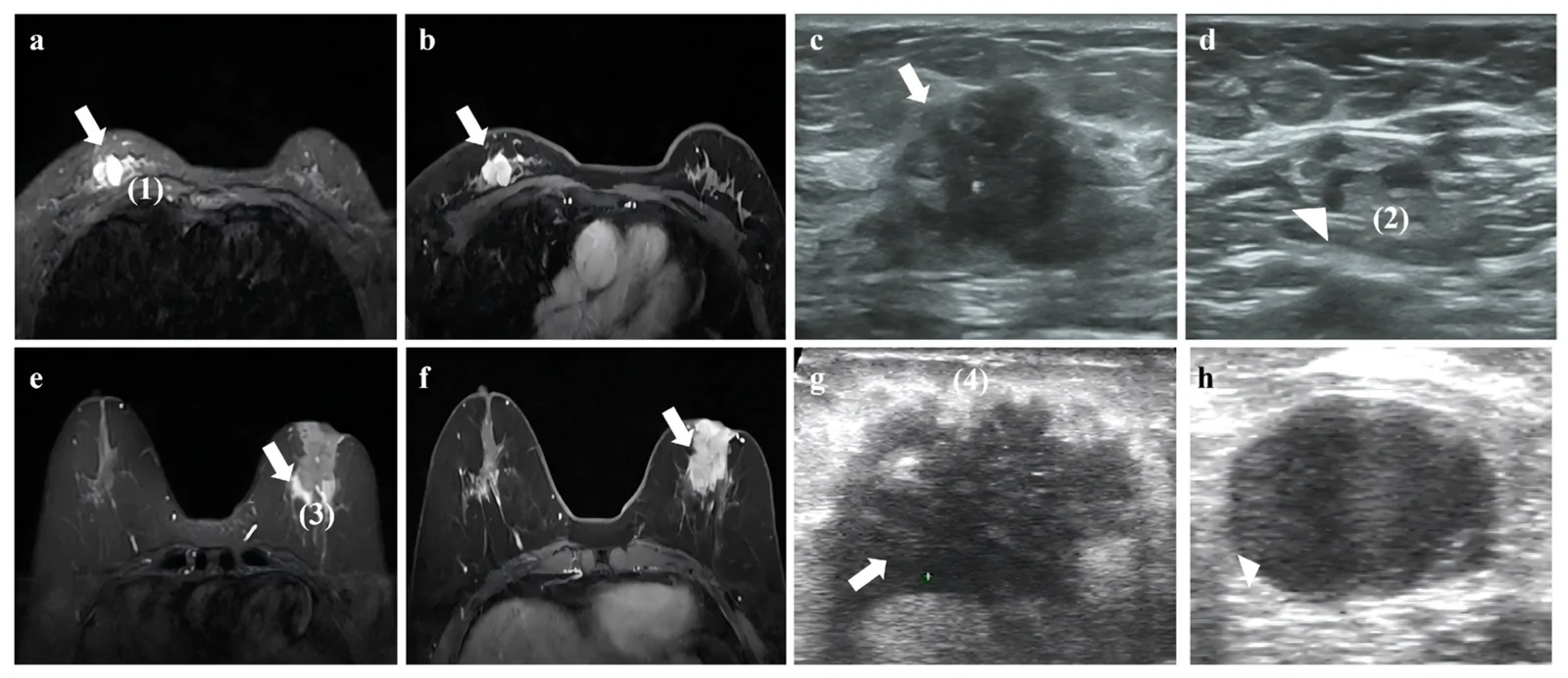
Examples of pretreatment MRI and US in two cN+ breast cancer patients. (**a**–**d**) A 59-year-old woman (cT2, HER2-positive subtype, Ki-67 15%) achieved ypN0 after NAC (model: ypN0). (**a**) T2WI shows the primary tumor with intratumoral T2 hyperintensity (1); T2 signal intensity, hyperintense. (**b**) DCE-MRI demonstrates lesion enhancement. (**c**) US shows a parallel-oriented hypoechoic mass. (**d**) Axillary US shows the target lymph node with a preserved fatty hilum (2); hilum, present. (**e**–**h**) A 69-year-old woman (cT2, HR+/HER2− subtype, Ki-67 30%) had residual nodal disease (ypN1–3) after NAC (model: ypN1–3). (**e**) T2WI shows peritumoral edema adjacent to the primary tumor (3); peritumoral edema, present. (**f**) DCE-MRI demonstrates lesion enhancement. (**g**) US shows an echogenic rind (4); echogenic rind, present. (**h**) Axillary US shows the target lymph node. Arrows indicate the primary tumor and arrowheads indicate the target lymph node; (1)–(4) denote the corresponding imaging descriptors.

Using the training-cohort Youden index–derived cutoff, the MT_UTA + Clinical model achieved sensitivities of 0.731, 0.680, and 0.944 and specificities of 0.904, 0.912, and 0.750 in the training, internal validation, and external validation cohorts, respectively. Accuracy was 0.842, 0.814, and 0.842 in the three cohorts, and the negative predictive values were 0.859, 0.795, and 0.938, respectively (Table 4). MT_UTA + Clinical also showed the lowest Brier scores across cohorts, with values of 0.128, 0.161, and 0.104, respectively, and higher net benefit than either Clinical or MT_UTA across most threshold probabilities (Figure 5; Supplementary Table S13). Representative pretreatment MRI and US findings in patients with ypN0 and ypN1–3 are shown in Figure 7.

## 4. Discussion

In this multicenter cohort of cN+ breast cancer treated with NAC, we compared pretreatment descriptor-based models derived from MRI tumor, US tumor, and US axillary assessments for predicting ypN0. Three main findings emerged. First, MRI tumor descriptors provided the strongest unimodal imaging signal. Second, adding US tumor and axillary descriptors provided complementary but modest incremental information beyond MRI tumor descriptors. Third, integration with clinicopathologic variables yielded the best overall performance across the training, internal validation, and external validation cohorts. These findings support a cross-anatomic, descriptor-based strategy for pretreatment ypN0 risk stratification, while underscoring the need for larger prospective validation before clinical implementation.

### 4.1. Comparison with Previous Studies and Modality-Specific Interpretation

In the single-modality comparison, the MT model achieved AUCs of 0.761, 0.716, and 0.769 in the training, internal validation, and external validation cohorts, respectively, indicating that baseline MRI tumor phenotypes capture substantial information relevant to ypN0. From an imaging standpoint, DCE-MRI provides a comprehensive depiction of primary tumor morphology and peritumoral findings (e.g., spatial distribution and peritumoral edema), which may contribute to this discriminatory signal [36]. At the same time, conventional breast MRI has inherent constraints for axillary assessment: variations in axillary coverage, spatial resolution, and scanning protocols can limit reliable recognition of fine nodal structures such as cortical thickness and the nodal hilum [16]. These complementary strengths and limitations motivate a cross-anatomic strategy in which MRI primarily characterizes the breast tumor while US provides pragmatic nodal morphologic assessment under routine workflows.

Prior MRI–US-based models have reported acceptable performance for post-NAC axillary response prediction. Kim et al. reported AUCs of 0.84 and 0.78 in the test and validation cohorts, respectively, using MRI, US, and clinicopathologic variables [17]. Malhaire et al. used baseline MRI and axillary US descriptors with biologic factors and achieved AUCs of 0.860 and 0.843 in the training and test sets, respectively [18]. In addition, a multimodal deep-learning model based on US, MRI, and clinical parameters achieved AUCs of 0.819 and 0.809 for ALN metastasis prediction in internal and external validation cohorts [19]. More recently, Cheng et al. reported a multimodal nomogram combining clinical and imaging features for predicting axillary pathologic complete response in a comparable cN+ population [20]. In our study, the MT_UTA + Clinical model achieved AUCs of 0.858 and 0.931 in the internal and external validation cohorts, respectively. Direct ranking across studies is inappropriate because endpoint definitions, imaging time points, feature types, cohort composition, and validation designs differ.

Exploratory subgroup analyses suggested generally preserved discrimination across molecular subtypes and cN stages, but the small number of patients in several subgroups, particularly triple-negative disease, precluded definitive subtype-specific conclusions (Supplementary Table S14). A key distinction from prior work is that we did not assume that more modalities are necessarily better. Instead, MT, UT, and UA were treated as three routinely available feature modules and compared head-to-head within the same cN+ cohort under a unified modeling scheme. This design allowed us to separate the contribution of modality from that of anatomic region and to clarify whether US tumor or axillary descriptors added meaningful information beyond MRI tumor descriptors. Our findings suggest that MRI tumor descriptors provided the dominant pretreatment imaging signal, whereas US descriptors offered complementary but modest incremental information. Compared with radiomics or deep-learning pipelines that require quantitative image processing, the present descriptor-based approach may be more readily aligned with structured clinical reporting.

### 4.2. Incremental Value of Multimodality Integration

In this head-to-head framework, US-related gains were more evident in reclassification metrics than in AUC. Under the fixed cutoff from the training cohort, UT- and UA-containing models showed distinct sensitivity–specificity trade-offs, with UT combinations yielding higher sensitivity and UA combinations yielding higher specificity. In the internal and external validation cohorts, US-containing models showed modest AUC gains over MT, with selected NRI improvements observed in the external validation cohort. These results suggest that US may contribute complementary information by reshaping risk estimates, but the incremental benefit appears modest and requires confirmation in larger validation cohorts.

### 4.3. Potential Clinical Implications

The intended clinical role of the proposed model is pretreatment risk stratification rather than direct selection of axillary surgery. In current practice, decisions regarding SLNB, ALND, or axillary de-escalation still require post-NAC clinical assessment and pathologic confirmation. The clinicoradiologic model may instead help identify patients with a high predicted probability of ypN0 who could be considered for closer post-NAC imaging surveillance, multidisciplinary discussion, or enrollment in prospective axillary de-escalation studies. Conversely, a low predicted probability of ypN0 may alert clinicians to a higher likelihood of residual nodal disease and support more cautious preoperative axillary reassessment. In this context, the model is best viewed as a decision-support tool that complements, but does not replace, standard clinical and pathologic evaluation, and should not be used as a standalone basis to omit axillary staging or determine the extent of axillary surgery.

### 4.4. Interpretation of Selected Imaging and Clinical Predictors

The selected imaging features can be interpreted across three phenotypic dimensions: spatial distribution/heterogeneity, invasive growth/interface characteristics, and peritumoral microenvironment signs. On MRI, the presence of MF/MC, irregular shape, and indistinct/spiculated margins were associated with a lower probability of ypN0, while peritumoral edema was a negative predictor. These descriptors may partly reflect underlying clinicopathologic phenotypes, as supported by the supplementary association analysis. Hyperintense T2 signal was positively associated with ypN0 in the multivariable model. MF/MC reflects complex spatial distribution and heterogeneity, consistent with pathologic observations linking it to higher nodal involvement risk [37]. Irregular shape and indistinct margins suggest infiltrative growth. Peritumoral edema is linked to vascular permeability, lymphatic drainage, and stromal response, correlating with higher aggressiveness and axillary burden [38,39]. The association observed for T2 hyperintensity warrants caution because its histologic substrates may include necrosis, mucinous components, or increased stromal water content, and prior findings regarding its relationship with treatment response and prognosis have not been fully consistent [18,19]. Accordingly, T2 hyperintensity should be interpreted as a multivariable imaging phenotype rather than a single-mechanism surrogate.

On US, complementary information was captured from a primary tumor phenotype (UT) + axillary nodal structural phenotype (UA) perspective. For UA, the presence of a nodal hilum was associated with higher probability of ypN0, whereas for UT, non-parallel orientation and an echogenic rind were associated with lower probability of ypN0. The nodal hilum is commonly used as a marker of preserved nodal architecture and lower tumor replacement, consistent in direction with prior US-based assessments of axillary response [17,40]. UT signs may provide phenotypic information not fully overlapping with MRI descriptors; however, given operator and device dependence, they should be interpreted as phenotype-level signals rather than as direct indicators of a single pathologic process. Among clinical variables, cN stage and molecular subtype were independently associated with ypN0 in the training cohort. HER2-positive molecular subtype remained strongly associated with ypN0 in the final MT_UTA + Clinical model (OR, 9.93; 95% CI, 3.51–28.08; *p* < 0.001; Supplementary Table S12), together with selected imaging descriptors, including peritumoral edema, echogenic rind, and preserved nodal hilum. The relatively high proportion of HER2-positive tumors likely reflects the selected cN+ neoadjuvant-treatment population, in which HER2-positive disease is commonly represented because of its responsiveness to anti-HER2-based systemic therapy. This subtype composition should therefore be considered when interpreting model generalizability.

### 4.5. Limitations and Future Directions

Several limitations merit consideration. First, although this retrospective multicenter study included an independent external validation cohort, the overall sample size remained modest, particularly the external validation cohort and molecular subtype subgroups; the limited number of outcome events may still introduce model optimism. Prospective multicenter validation in larger cohorts is required to confirm robustness and generalizability. Second, molecular subtype composition may affect reproducibility, and external cohorts with more representative subtype distributions are needed. Third, the model was based on structured semantic descriptors rather than quantitative radiomics or deep-learning features; although this improves interpretability and facilitates integration into routine imaging reports, head-to-head comparison with radiomics or artificial intelligence models was not performed. Finally, MRI and US were acquired under routine clinical protocols, and only pretreatment imaging was evaluated. Serial imaging during NAC has been shown to provide additional predictive information [41]; future studies should assess whether optimized axillary imaging protocols, serial on-treatment imaging, and larger subtype-balanced cohorts can improve performance and clarify the clinical utility of US-related reclassification signals.

## 5. Conclusions

In conclusion, this head-to-head cross-anatomic analysis of routinely available pretreatment MRI and breast/axillary US descriptors provides a practical approach for preoperative risk stratification of post-NAC ypN0 in patients with cN+ breast cancer. MRI tumor descriptors showed the strongest discrimination among the imaging-based models, while US tumor and nodal descriptors contributed complementary information with modest incremental effects. The integrated clinicoradiologic model may support pretreatment risk assessment and multidisciplinary planning for post-NAC axillary reassessment and future TAD-based de-escalation studies, but should not be used as a standalone determinant of axillary surgery. Despite independent external validation, larger prospective multicenter studies are needed to confirm its broader clinical utility.

## Figures and Tables

**Table 1:** Baseline Clinicopathologic Characteristics and Primary Outcome of the Study Cohorts.

Characteristic	Total (*n* = 243)	Training Cohort (*n* = 146)	Internal Validation Cohort (*n* = 59)	External Validation Cohort (*n* = 38)	*p*
Age, years *	51.84 ± 10.67	51.77 ± 10.43	54.03 ± 11.45	48.71 ± 9.77	0.056
Menopausal status, *n* (%)					0.530
Premenopausal	102 (41.98)	60 (41.10)	23 (38.98)	19 (50.00)	
Postmenopausal	141 (58.02)	86 (58.90)	36 (61.02)	19 (50.00)	
cT stage, *n* (%)					0.014
cT1–2	180 (74.07)	112 (76.71)	47 (79.66)	21 (55.26)	
cT3–4	63 (25.93)	34 (23.29)	12 (20.34)	17 (44.74)	
cN stage, *n* (%)					0.127
cN1	117 (48.15)	67 (45.89)	26 (44.07)	24 (63.16)	
cN2–3	126 (51.85)	79 (54.11)	33 (55.93)	14 (36.84)	
Molecular subtype, *n* (%)					0.635
HR+/HER2−	114 (46.91)	73 (50.00)	23 (38.98)	18 (47.37)	
HER2-positive	102 (41.98)	57 (39.04)	28 (47.46)	17 (44.74)	
Triple-negative	27 (11.11)	16 (10.96)	8 (13.56)	3 (7.89)	
ER status, *n* (%)					0.621
Negative	87 (35.80)	51 (34.93)	24 (40.68)	12 (31.58)	
Positive	156 (64.20)	95 (65.07)	35 (59.32)	26 (68.42)	
PR status, *n* (%)					0.145
Negative	97 (39.92)	60 (41.10)	27 (45.76)	10 (26.32)	
Positive	146 (60.08)	86 (58.90)	32 (54.24)	28 (73.68)	
HER2 status, *n* (%)					0.506
Negative	141 (58.02)	89 (60.96)	31 (52.54)	21 (55.26)	
Positive	102 (41.98)	57 (39.04)	28 (47.46)	17 (44.74)	
Ki-67 index, *n* (%)					0.074
<20%	41 (16.87)	31 (21.23)	7 (11.86)	3 (7.89)	
≥20%	202 (83.13)	115 (78.77)	52 (88.14)	35 (92.11)	
Histologic type, *n* (%)					0.343
IC-NST	229 (94.24)	135 (92.47)	57 (96.61)	37 (97.37)	
ILC/other	14 (5.76)	11 (7.53)	2 (3.39)	1 (2.63)	
Histologic grade, *n* (%) †					0.177
Grade I	7 (2.88)	5 (3.42)	1 (1.69)	1 (2.63)	
Grade II	112 (46.09)	63 (43.15)	30 (50.85)	19 (50.00)	
Grade III	56 (23.05)	28 (19.18)	10 (16.95)	18 (47.37)	
Unknown	68 (27.98)	50 (34.25)	18 (30.51)	0 (0.00)	
Primary outcome, *n* (%)					0.350
ypN0	95 (39.09)	52 (35.62)	25 (42.37)	18 (47.37)	
ypN1–3	148 (60.91)	94 (64.38)	34 (57.63)	20 (52.63)	

Note—Unless otherwise indicated, data are numbers of patients, with percentages in parentheses. *p* values were calculated using one-way ANOVA for continuous variables and the χ^2^ test for categorical variables. cT = clinical tumor stage; cN = clinical nodal stage; ER = estrogen receptor; PR = progesterone receptor; HER2 = human epidermal growth factor receptor 2; HR = hormone receptor; IC-NST = invasive carcinoma of no special type; ILC = invasive lobular carcinoma; ypN = postneoadjuvant pathologic nodal stage. * Data are presented as mean ± standard deviation. † Histologic grade was unknown in 68 patients; the *p* value was calculated among the 175 patients with known grade.

**Table 2:** Pretreatment Imaging Characteristics in the Training Cohort by Postneoadjuvant Nodal Status.

Characteristic	Overall (*n* = 146)	ypN1–3 (*n* = 94)	ypN0 (*n* = 52)	*p*
MRI Characteristics				
MF/MC, *n* (%) †				0.054
Absent	77 (52.74)	44 (46.81)	33 (63.46)	
Present	69 (47.26)	50 (53.19)	19 (36.54)	
Depth localization, *n* (%) †				0.299
Anterior third	34 (23.29)	21 (22.34)	13 (25.00)	
Middle third	79 (54.11)	48 (51.06)	31 (59.62)	
Posterior third	33 (22.60)	25 (26.60)	8 (15.38)	
Morphology, *n* (%)				0.445
Mass	131 (89.73)	83 (88.30)	48 (92.31)	
NME	15 (10.27)	11 (11.70)	4 (7.69)	
FGT, *n* (%)				0.618
Fatty/scattered	43 (29.45)	29 (30.85)	14 (26.92)	
Heterogeneous/extremely dense	103 (70.55)	65 (69.15)	38 (73.08)	
BPE, *n* (%)				0.744
Minimal/mild	109 (74.66)	71 (75.53)	38 (73.08)	
Moderate/marked	37 (25.34)	23 (24.47)	14 (26.92)	
Shape, *n* (%) †				0.126
Oval/lobulated/round	22 (15.07)	11 (11.70)	11 (21.15)	
Irregular	124 (84.93)	83 (88.30)	41 (78.85)	
Margin, *n* (%) †				0.008
Circumscribed	28 (19.18)	12 (12.77)	16 (30.77)	
Indistinct/spiculated	118 (80.82)	82 (87.23)	36 (69.23)	
Internal enhancement characteristics, *n* (%)				0.439
Homogeneous	7 (4.79)	4 (4.26)	3 (5.77)	
Heterogeneous	125 (85.62)	83 (88.30)	42 (80.77)	
Thick rim enhancement	14 (9.59)	7 (7.45)	7 (13.46)	
T2 signal intensity, *n* (%) †				0.065
Not hyperintense	133 (91.10)	89 (94.68)	44 (84.62)	
Hyperintense	13 (8.90)	5 (5.32)	8 (15.38)	
Peritumoral edema, *n* (%) †				<0.001
Absent	49 (33.56)	21 (22.34)	28 (53.85)	
Present	97 (66.44)	73 (77.66)	24 (46.15)	
Enhancement kinetics, *n* (%)				0.465
Persistent/plateau	110 (75.34)	69 (73.40)	41 (78.85)	
Washout	36 (24.66)	25 (26.60)	11 (21.15)	
US Tumor Characteristics				
Orientation, *n* (%) †				0.017
Parallel	120 (82.19)	72 (76.60)	48 (92.31)	
Non-parallel	26 (17.81)	22 (23.40)	4 (7.69)	
Shape, *n* (%)				0.153
Oval/lobulated/round	35 (23.97)	19 (20.21)	16 (30.77)	
Irregular	111 (76.03)	75 (79.79)	36 (69.23)	
Margin, *n* (%)				0.721
Circumscribed	9 (6.16)	5 (5.32)	4 (7.69)	
Indistinct/angular/microlobulated/spiculated	137 (93.84)	89 (94.68)	48 (92.31)	
Echo pattern, *n* (%)				0.442
Hypoechoic	82 (56.16)	55 (58.51)	27 (51.92)	
Heterogeneous/mixed solid and cystic	64 (43.84)	39 (41.49)	25 (48.08)	
Posterior features, *n* (%)				0.962
No posterior features	35 (23.97)	22 (23.40)	13 (25.00)	
Enhancement	18 (12.33)	12 (12.77)	6 (11.54)	
Shadowing	93 (63.70)	60 (63.83)	33 (63.46)	
Echogenic rind, *n* (%) †				<0.001
Absent	68 (46.58)	34 (36.17)	34 (65.38)	
Present	78 (53.42)	60 (63.83)	18 (34.62)	
Calcifications in a mass, *n* (%)				0.864
Absent	27 (18.49)	17 (18.09)	10 (19.23)	
Present	119 (81.51)	77 (81.91)	42 (80.77)	
US Axillary Lymph Node Characteristics				
Ratio of long/short axis, *n* (%)				0.527
<2	92 (63.01)	61 (64.89)	31 (59.62)	
≥2	54 (36.99)	33 (35.11)	21 (40.38)	
Margin, *n* (%)				0.567
Circumscribed	86 (58.90)	57 (60.64)	29 (55.77)	
Non-circumscribed	60 (41.10)	37 (39.36)	23 (44.23)	
Cortical thickening, *n* (%)				0.583
No/uniform	94 (64.38)	59 (62.77)	35 (67.31)	
Non-uniform	52 (35.62)	35 (37.23)	17 (32.69)	
Hilum, *n* (%) †				<0.001
Absent	108 (73.97)	79 (84.04)	29 (55.77)	
Present	38 (26.03)	15 (15.96)	23 (44.23)	

Note—Unless otherwise indicated, data are numbers of patients, with percentages in parentheses. BPE = background parenchymal enhancement; FGT = fibroglandular tissue; MF/MC = multifocal and/or multicentric disease; NME = nonmass enhancement; US = ultrasound. † Descriptor retained by the training-cohort LASSO procedure.

**Table 3:** Univariate and Multivariate Analyses for Prediction of ypN0 in the Training Cohort.

Variables	Univariable Analysis		Multivariable Analysis	
	OR (95% CI)	*p*	OR (95% CI)	*p*
Age, per 1-year increase	0.99 (0.96–1.03)	0.737		
Menopausal status, postmenopausal vs. premenopausal	0.93 (0.47–1.84)	0.825		
cT stage, cT3–4 vs. cT1–2	0.98 (0.44–2.19)	0.964		
cN stage, cN2–3 vs. cN1	0.48 (0.24–0.95)	0.035	0.44 (0.20–0.96)	0.040
Molecular subtype †	—	<0.001	—	<0.001
HER2-positive vs. HR+/HER2−	8.71 (3.84–19.79)	<0.001	9.03 (3.90–20.90)	<0.001
Triple-negative vs. HR+/HER2−	1.69 (0.47–6.15)	0.423	1.72 (0.46–6.37)	0.417
ER status, positive vs. negative	0.41 (0.20–0.84)	0.014		
PR status, positive vs. negative	0.44 (0.22–0.88)	0.021		
HER2 status, positive vs. negative	7.82 (3.65–16.78)	<0.001		
Ki-67 index, ≥20% vs. <20%	2.20 (0.88–5.54)	0.093		
Histologic type, ILC/other vs. IC-NST ‡	Not estimable	—		
Histologic grade, Grade III vs. Grade I–II §	0.70 (0.26–1.88)	0.477		

Note—Data are odds ratios (ORs), with 95% CIs in parentheses. OR > 1 indicates greater odds of ypN0. cT = clinical tumor stage; cN = clinical nodal stage; ER = estrogen receptor; PR = progesterone receptor; HER2 = human epidermal growth factor receptor 2; HR = hormone receptor; IC-NST = invasive carcinoma of no special type; ILC = invasive lobular carcinoma. † For molecular subtype, the overall Wald *p* value is shown in the parent row; HR+/HER2− is the reference category. ‡ The univariable OR for histologic type was not meaningfully estimable because of separation. § Histologic grade was available in 96 patients; unknown grades were excluded, and Grades I–II were the reference category.

**Table 4:** Performance of Models for Predicting ypN0 in the Training and Validation Cohorts.

Cohort	Model †	Cutoff *	AUC (95% CI)	SEN	SPE	PPV	NPV	ACC
Training cohort	MT	0.284	0.761 (0.679–0.843)	0.827	0.574	0.518	0.857	0.664
	UT	0.400	0.673 (0.589–0.756)	0.635	0.691	0.532	0.774	0.671
	UA	0.437	0.641 (0.564–0.719)	0.442	0.840	0.605	0.731	0.699
	MT_UT	0.383	0.804 (0.729–0.880)	0.731	0.777	0.644	0.839	0.760
	MT_UA	0.456	0.794 (0.714–0.873)	0.596	0.862	0.705	0.794	0.767
	UT_UA	0.343	0.717 (0.632–0.802)	0.692	0.628	0.507	0.787	0.651
	MT_UTA	0.354	0.817 (0.741–0.893)	0.750	0.766	0.639	0.847	0.760
	Clinical	0.282	0.783 (0.707–0.859)	0.750	0.734	0.609	0.841	0.740
	MT_UTA + Clinical	0.467	0.886 (0.832–0.941)	0.731	0.904	0.809	0.859	0.842
Internal validation cohort	MT	—	0.716 (0.585–0.848)	0.840	0.588	0.600	0.833	0.695
	UT	—	0.619 (0.483–0.755)	0.560	0.676	0.560	0.676	0.627
	UA	—	0.606 (0.493–0.720)	0.360	0.853	0.643	0.644	0.644
	MT_UT	—	0.754 (0.631–0.876)	0.480	0.912	0.800	0.705	0.729
	MT_UA	—	0.753 (0.629–0.877)	0.920	0.500	0.575	0.895	0.678
	UT_UA	—	0.639 (0.496–0.781)	0.600	0.618	0.536	0.677	0.610
	MT_UTA	—	0.761 (0.638–0.883)	0.520	0.912	0.812	0.721	0.746
	Clinical	—	0.788 (0.672–0.904)	0.840	0.735	0.700	0.862	0.780
	MT_UTA + Clinical	—	0.858 (0.763–0.953)	0.680	0.912	0.850	0.795	0.814
External validation cohort	MT	—	0.769 (0.620–0.919)	0.944	0.400	0.586	0.889	0.658
	UT	—	0.656 (0.502–0.809)	0.722	0.550	0.591	0.688	0.632
	UA	—	0.675 (0.532–0.818)	0.500	0.850	0.750	0.654	0.684
	MT_UT	—	0.792 (0.650–0.934)	0.833	0.600	0.652	0.800	0.711
	MT_UA	—	0.779 (0.628–0.930)	0.556	0.800	0.714	0.667	0.684
	UT_UA	—	0.740 (0.586–0.895)	0.778	0.550	0.609	0.733	0.658
	MT_UTA	—	0.806 (0.667–0.944)	0.667	0.600	0.600	0.667	0.632
	Clinical	—	0.831 (0.697–0.964)	0.778	0.800	0.778	0.800	0.789
	MT_UTA + Clinical	—	0.931 (0.856–1.000)	0.944	0.750	0.773	0.938	0.842

Note—Data in parentheses are 95% CIs. AUC = area under the receiver operating characteristic curve; ACC = accuracy; NPV = negative predictive value; PPV = positive predictive value; SEN = sensitivity; SPE = specificity; MT = MRI tumor; UT = ultrasound tumor; UA = ultrasound axilla. * Cutoffs were determined in the training cohort using the Youden index and applied unchanged to both validation cohorts. † MT_UT, MT_UA, UT_UA, and MT_UTA denote the corresponding combinations of feature sets; Clinical denotes the clinical-only model; MT_UTA + Clinical denotes the clinicoradiologic model.

**Table 5:** Incremental Value of US Descriptors Beyond the MRI Tumor Model for Predicting ypN0.

Cohort	Model Comparison †	ΔAUC	*p*	NRI (95% CI)	*p*	IDI (95% CI)	*p*
Training cohort	MT_UT vs. MT	0.044	0.070	0.631 (0.285–0.938)	<0.001	0.059 (0.018–0.095)	0.003
	MT_UA vs. MT	0.033	0.167	0.566 (0.232–0.864)	<0.001	0.065 (0.022–0.108)	0.003
	MT_UTA vs. MT	0.056	0.045	0.606 (0.277–0.909)	<0.001	0.102 (0.049–0.151)	<0.001
Internal validation cohort	MT_UT vs. MT	0.037	0.378	0.452 (−0.066–0.948)	0.077	0.028 (−0.029–0.084)	0.320
	MT_UA vs. MT	0.036	0.270	0.426 (−0.012–0.864)	0.058	0.058 (−0.002–0.122)	0.069
	MT_UTA vs. MT	0.045	0.331	0.414 (−0.099–0.911)	0.105	0.069 (−0.013–0.151)	0.089
External validation cohort	MT_UT vs. MT	0.022	0.620	0.544 (−0.078–1.155)	0.072	0.036 (−0.036–0.106)	0.317
	MT_UA vs. MT	0.010	0.786	0.700 (0.156–1.233)	0.012	0.062 (−0.009–0.133)	0.088
	MT_UTA vs. MT	0.036	0.463	0.644 (0.022–1.189)	0.032	0.073 (−0.019–0.165)	0.122

Note—ΔAUC indicates the absolute increase in AUC relative to MT; NRI and IDI are continuous measures. *p* values correspond to the respective between-model comparisons. AUC = area under the receiver operating characteristic curve; IDI = integrated discrimination improvement; NRI = net reclassification improvement; MT = MRI tumor; UT = US tumor; UA = US axilla. † MT_UT, MT_UA, and MT_UTA denote the corresponding combinations of feature sets.

**Table 6:** Incremental Value of the Clinicoradiologic Model Beyond Imaging-Only and Clinical-Only Models for Predicting ypN0.

Cohort	Model Comparison †	ΔAUC	*p* Value	NRI (95% CI)	*p* Value	IDI (95% CI)	*p* Value
Training cohort	MT_UTA + Clinical vs. MT_UTA	0.070	0.010	0.938 (0.626–1.228)	<0.001	0.133 (0.074–0.191)	<0.001
	MT_UTA + Clinical vs. Clinical	0.103	0.003	1.036 (0.737–1.313)	<0.001	0.208 (0.131–0.281)	<0.001
Internal validation cohort	MT_UTA + Clinical vs. MT_UTA	0.097	0.046	0.991 (0.536–1.424)	<0.001	0.153 (0.061–0.242)	<0.001
	MT_UTA + Clinical vs. Clinical	0.071	0.170	0.607 (0.132–1.066)	0.010	0.156 (0.046–0.261)	0.005
External validation cohort	MT_UTA + Clinical vs. MT_UTA	0.125	0.041	1.256 (0.722–1.689)	<0.001	0.204 (0.087–0.307)	<0.001
	MT_UTA + Clinical vs. Clinical	0.100	0.099	0.844 (0.245–1.378)	0.003	0.231 (0.080–0.392)	0.003

Note—ΔAUC indicates the absolute increase in AUC for MT_UTA + Clinical relative to the comparator model; NRI and IDI are continuous measures. *p* values correspond to the respective between-model comparisons. AUC = area under the receiver operating characteristic curve; IDI = integrated discrimination improvement; NRI = net reclassification improvement. † Clinical includes clinical N stage and molecular subtype; MT_UTA comprises MRI tumor, US tumor, and US axillary descriptors.

## Data Availability

The data presented in this study are not publicly available due to patient privacy, ethical restrictions, and institutional data protection regulations. De-identified data may be available from the corresponding author upon reasonable request, subject to approval by the relevant ethics committees and participating institutions.

## Supplementary Materials

The following supporting information can be downloaded at: https://www.mdpi.com/article/10.3390/diagnostics16172853/s1, Supplementary Methods. Neoadjuvant Systemic Therapy Regimens; Table S1. MRI scan sequences and parameters; Table S2. Definitions and classification of MRI descriptors; Table S3. Definitions and classification of ultrasound descriptors for the primary breast tumor; Table S4. Ultrasound descriptors of axillary lymph nodes; Table S5. Definitions of clinicopathologic variables and neoadjuvant systemic therapy regimens; Table S6. Neoadjuvant systemic therapy and postneoadjuvant pathologic characteristics across cohorts; Table S7. Inter-reader agreement of structured MRI and ultrasound imaging descriptors; Table S8. Distribution of candidate imaging descriptors between the training and internal validation cohorts; Table S9. Distribution of LASSO-selected imaging descriptors across cohorts; Table S10. LASSO-selected imaging descriptors and corresponding coefficients for predicting ypN0; Table S11. Associations between LASSO-selected imaging descriptors and clinicopathologic variables; Table S12. Full coefficient specification of the Clinical and MT_UTA + Clinical models; Table S13. Discrimination and quantitative calibration of the Clinical, MT_UTA, and MT_UTA + Clinical models across cohorts; Table S14. Exploratory subgroup performance of the imaging, clinical, and integrated models in the pooled validation cohort; Table S15. Exploratory associations of clinicopathologic factors with ypCR and ypT0/is; Table S16. Neoadjuvant systemic therapy regimens by molecular subtype and cohort; Figure S1. LASSO cross-validation error. Figure S2. LASSO coefficient path. Figure S3. Association heatmap between LASSO-selected imaging descriptors and clinicopathologic variables. References [23,24,25,26,27,28,29,30,31,32,33,34,35,42,43,44,45] are cited in the Supplementary Materials.

## Abbreviations

The following abbreviations are used in this manuscript:

ALN	axillary lymph node
ALND	axillary lymph node dissection
AUC	area under the receiver operating characteristic curve
BI-RADS	Breast Imaging Reporting and Data System
cN	clinical nodal stage
cT	clinical tumor stage
DCE-MRI	dynamic contrast-enhanced magnetic resonance imaging
ER	estrogen receptor
HER2	human epidermal growth factor receptor 2
HR	hormone receptor
IDI	integrated discrimination improvement
LASSO	least absolute shrinkage and selection operator
MRI	magnetic resonance imaging
MT	MRI tumor descriptor model
MT_UA	combination of MRI tumor and US axilla descriptors
MT_UT	combination of MRI tumor and US tumor descriptors
MT_UTA	combination of MRI tumor, US tumor, and US axilla descriptors
NAC	neoadjuvant chemotherapy
NPV	negative predictive value
NRI	net reclassification improvement
PPV	positive predictive value
PR	progesterone receptor
SLNB	sentinel lymph node biopsy
TAD	targeted axillary dissection
UA	US axilla descriptor model
US	ultrasound
UT	US tumor descriptor model
UT_UA	combination of US tumor and US axilla descriptors
ypN0	postneoadjuvant pathologic node-negative status
