# Supplementary material for: Pretreatment MRI and Ultrasound Features of the Primary Tumor and Axillary Nodes for Predicting Axillary Pathologic Complete Response After Neoadjuvant Chemotherapy: A Cross-Anatomic Comparison
Source: Diagnostics (Basel). 2026 Sep 4;16(17):2853. doi: 10.3390/diagnostics16172853 (PMC13565590; doi:10.3390/diagnostics16172853)
Supplement: Supplementary file 1 [file diagnostics-16-02853-s001.zip › diagnostics-4501147-supplementary.pdf]

Table S1. MRI scan sequences and parameters

	United Imaging 1.5T (uMR 560)	Philips 1.5T (Achieva)	GE 3.0T (Discovery 750)	Siemens 1.5 T (MAGNETOM Aera)
**Sequence**	DCE	DCE	DCE	DCE
**TR (ms)**	5.1	7	3.8	8.6
**TE (ms)**	2.1	3.4	1.6	4.7
**FOV (mm^2)**	320×320	340×340	360×360	340 × 340
**Slice thickness (mm)**	2.4	3	1.4	2.5
**Acquisition time (sec)**	394	568	737	455

TR, repetition time; TE, echo time; FOV, field of view; DCE, dynamic contrast-enhanced.

Table S2. Definitions and classification of MRI descriptors

Descriptor	Categories	Definition	Reference Source
**Morphology**	Mass; Nonmass enhancement (NME)	Mass: a space-occupying lesion with convex-outward margins; NME: enhancement without a distinct space-occupying form.	
**Shape**	Oval/Lobulated/Round ; Irregular	Determined by lesion contour on axial postcontrast images.	
**Margin**	Circumscribed; Indistinct/Spiculated	Circumscribed: well-defined interface with adjacent tissue; Indistinct/Spiculated: blurred or radiating margins.	[1, 2]
**Internal enhancement characteristics**	Homogeneous; Heterogeneous; Thick rim enhancement	Characterized by the distribution and uniformity of enhancement within the lesion.	
**T2 signal intensity**	Hyperintense; Not hyperintense	Hyperintense: uniformly bright signal throughout the lesion (i.e., "lymph node bright").	
**Peritumoral edema**	Absent; Present	Present when high T2 signal intensity is observed in the peritumoral soft tissues, approaching that of water.	[3]
**Enhancement kinetics**	Persistent/Plateau; Washout	Assessed on DCE MRI using time–intensity curves (persistent/plateau or washout pattern).	[1, 2]
**Depth localization**	Anterior third; Middle third; Posterior third	Determined according to the lesion's location within the anteroposterior	

		depth of the breast.	
**Multifocal/multicentric (MF/MC) disease**	Absent; Present	Defined as ≥2 distinct lesions within the ipsilateral breast: multifocal in the same quadrant, multicentric in different quadrants.	[3]
**Fibroglandular tissue (FGT)**	Fatty/Scattered; Heterogeneous/Extremely dense	Classified according to the amount of fibroglandular tissue on MRI.	[1, 2]
**Background parenchymal enhancement (BPE)**	Minimal/Mild; Moderate/Marked	Classified according to the degree of background parenchymal enhancement on DCE MRI.	

Table S3. Definitions and classification of ultrasound descriptors for the primary breast tumor

Descriptor	Categories	Definition	Reference Source
**Orientation**	Parallel; Non-parallel	Evaluated on the image plane orthogonal to the local skin line. Parallel: wider-than-tall with the long axis approximately parallel to the skin; Non-parallel: taller-than-wide with the long axis oriented toward the chest wall (vertical or oblique).	[1, 2]
**Shape**	Oval/Lobulated/Round; Irregular	Determined by lesion contour on the plane of maximal dimension.	
**Margin**	Circumscribed; Indistinct/Angular/Microlobulated/Spiculated	Circumscribed: well-defined interface; Non-circumscribed margins include indistinct, angular, microlobulated, or spiculated contours.	
**Echo pattern**	Hypoechoic; Heterogeneous/Mixed solid and cystic	Characterized by echogenicity relative to adjacent fibroglandular tissue. Hypoechoic: lower echogenicity than surrounding parenchyma;	

		Heterogeneous/Mixed: variable echogenicity or coexistence of solid and cystic components.
**Posterior features**	No posterior features; Enhancement; Shadowing	Assessed relative to adjacent parenchyma. Enhancement: increased through-transmission; Shadowing: posterior acoustic attenuation.
**Echogenic rind**	Absent; Present	A thick band of echogenic tissue surrounding all or part of a breast mass.
**Calcifications in a mass**	Absent; Present	Echogenic foci within a mass (typically within the solid component), with or without posterior acoustic shadowing.

Table S4. Ultrasound descriptors of axillary lymph nodes

Descriptor	Categories	Definition	Reference Source
**Ratio of long/short axis**	<2 vs ≥2	Long and short axes were measured on the longitudinal plane. The long-to-short axis (L/S) ratio was calculated as long axis ÷ short axis.	[4, 5]
**Lymph-node margin**	Circumscribed vs Non-circumscribed	Margin was assessed on gray-scale ultrasound. A circumscribed margin was defined as a smooth, well-defined, sharply demarcated outline. Any indistinct, blurred, angulated, lobulated, or spiculated border was classified as non-circumscribed.	[4, 6]
**Cortical thickening**	No/Uniform vs Non-uniform	Cortical thickness was measured at the thickest point on the longitudinal plane. Symmetric cortex ≤3 mm without focal bulging was coded as no/uniform. Focal or eccentric bulging, asymmetric thickening, or cortex equal to or thicker than the medulla (C/M ratio ≥1; typically >3 mm) was coded as non-uniform.	[4, 5]
**Hilum**	Absent vs Present	The echogenic hilum was defined as the central echogenic "nodal-core" structure (hilum–medulla complex) continuous with adjacent perinodal fat. Clear visualization was coded as present; complete loss or marked effacement was coded as absent.	[4, 6]

Supplementary Table S5. Definitions of Clinicopathologic Variables and Neoadjuvant Systemic Therapy Regimens

Variable	Definition	Reference Source
**Estrogen receptor (ER)**	Positive: ≥1% tumor nuclei staining<br>Negative: <1%	[7]
**Progesterone receptor (PR)**	Positive: ≥1% tumor nuclei staining<br>Negative: <1%	
**HER2 status**	Negative: IHC 0 or 1+<br>Equivocal: IHC 2+ → requires FISH<br>Positive: IHC 3+ or FISH amplified	[8]
**Ki-67 index**	Low: <20%<br>High: ≥20%	[9]

**Molecular subtype**	HR+/HER2−: ER and/or PR positive, HER2 negative<br>HER2-positive: HER2 positive, regardless of ER/PR status<br>Triple-negative: ER negative, PR negative, and HER2 negative	[10]
**Neoadjuvant systemic therapy regimen**	Anthracycline- and taxane-based chemotherapy: chemotherapy containing both an anthracycline (doxorubicin, epirubicin, or pirarubicin) and a taxane (paclitaxel, docetaxel, or nab-paclitaxel), given sequentially or in combination (eg, AC→T, TAC, dose-dense AC→P).	[11]
	Dual HER2-targeted therapy + chemotherapy: chemotherapy combined with two concurrent HER2-targeted agents, typically trastuzumab plus pertuzumab (eg, THP or TCHP).	[12]
	Single HER2-targeted therapy + chemotherapy: chemotherapy combined with one HER2-targeted agent (trastuzumab alone), without a second HER2-directed antibody.	[13]
	Chemotherapy + immunotherapy: chemotherapy combined with an immune checkpoint inhibitor (PD-1/PD-L1 antibody, eg, pembrolizumab), predominantly in triple-negative breast cancer.	[14]
	Other chemotherapy regimens: mainly taxane- and platinum-based, single-class, and capecitabine-containing regimens.	–
**Lymphovascular invasion**	Absent: no tumor cell emboli identified within endothelium-lined lymphatic or blood vessels in the tumor or peritumoral tissue on H&E sections (with D2-40/CD31/CD34 immunostaining when required).<br>Present: tumor cell clusters present within lymphatic or vascular spaces within or adjacent to the tumor bed; treatment-related fibrosis or hemorrhage without intraluminal tumor cells is not regarded as LVI.	[15]

**ypT stage**	ypT0/is: no residual invasive carcinoma in the breast; ypT0 = no residual invasive carcinoma or DCIS, ypTis = no residual invasive carcinoma but DCIS present.<br>ypT1–4: residual invasive carcinoma sized by the largest contiguous invasive focus (treatment-related fibrosis excluded): ypT1 ≤20 mm; ypT2 >20–50 mm; ypT3 >50 mm; ypT4 invasion of chest wall and/or skin.	[16]
**ypN stage**	ypN0: no metastatic carcinoma in resected regional lymph nodes; isolated tumor cells (≤0.2 mm or ≤200 cells) are recorded as ypN0(i+).<br>ypN1: metastasis (micrometastasis >0.2–2 mm or macrometastasis >2 mm) in 1–3 ipsilateral axillary lymph nodes and/or clinically occult internal mammary micrometastases.<br>ypN2: metastasis in 4–9 ipsilateral axillary lymph nodes, or clinically apparent internal mammary metastasis in the absence of axillary nodal metastasis.<br>ypN3: metastasis in ≥10 ipsilateral axillary nodes, infraclavicular nodal metastasis, specified internal mammary/axillary combinations, or ipsilateral supraclavicular nodal metastasis.	[16]
**Overall pathologic complete response**	Non-ypCR: residual invasive carcinoma present in the breast and/or regional lymph nodes.<br>ypCR: absence of residual invasive carcinoma in both the breast and regional lymph nodes (ypT0/is ypN0); residual DCIS is permitted.	[17]

Note.—DCIS = ductal carcinoma in situ; FISH = fluorescence in situ hybridization; HER2 = human epidermal growth factor receptor 2; HR = hormone receptor; IHC = immunohistochemistry; LVI = lymphovascular invasion; ypCR = overall pathologic complete response.

Supplementary Methods. Neoadjuvant Systemic Therapy Regimens

1. General principles

Neoadjuvant systemic therapy regimens were selected according to contemporary guideline recommendations, molecular subtype, tumor and nodal burden, comorbidities, treatment tolerance, and multidisciplinary clinical assessment. Regimens were categorized as anthracycline- and taxane-based chemotherapy, dual HER2-targeted therapy combined with chemotherapy, single HER2-targeted therapy combined with chemotherapy, chemotherapy combined with immunotherapy, and other chemotherapy regimens.

For HR+/HER2− tumors, neoadjuvant treatment mainly consisted of anthracycline- and taxane-based chemotherapy or individualized chemotherapy regimens according to clinical indication. For HER2-positive tumors, chemotherapy was combined with anti-HER2 targeted therapy, including trastuzumab with or without pertuzumab. For triple-negative tumors, anthracycline/taxane- or taxane/platinum-based chemotherapy was used, with immune checkpoint inhibitor–based chemoimmunotherapy administered in selected patients.

Standard regimen schedules, planned dose density, planned dose intensity, and cycle numbers were based on guideline-recommended or trial-supported protocols. Actual dose reductions, treatment delays, regimen modifications, or discontinuations were determined according to toxicity, comorbidities, treatment tolerance, and the treating physician's judgment. The distribution of regimen categories across cohorts is provided in Supplementary Table S6, and subtype-specific regimen distributions are provided in Supplementary Table S16.

2. Standard regimen schedules by molecular subtype

Molecular subtype	Common regimen category	Representative regimen	Planned dose density / intensity	Planned cycles
HR+/HER2−	Anthracycline- and taxane-based chemotherapy	AC/EC followed by taxane, or TAC	q2w/q3w depending on protocol; standard-dose anthracycline and taxane-based chemotherapy	6–8 cycles
HER2-positive	Chemotherapy + anti-HER2 therapy	THP, TCHP, or AC/EC followed by taxane + trastuzumab ± pertuzumab	q3w HER2-targeted therapy with taxane/platinum- or anthracycline-taxane-based chemotherapy	6–8 cycles
Triple-negative	Anthracycline/taxane- or taxane/platinum-based chemotherapy ± immunotherapy	AC/EC→T, TCb, or pembrolizumab-containing chemoimmunotherapy in selected patients	q2w/q3w chemotherapy; pembrolizumab-based regimens when clinically indicated	6–8 cycles, or protocol-based cycles for immunotherapy regimens
Other / individualized	Modified chemotherapy or endocrine-based therapy in selected patients	Capecitabine-containing, taxane/platinum, or individualized regimens	Modified according to tolerance/comorbidity	Individualized

Supplementary Table S6. Neoadjuvant Systemic Therapy and Postneoadjuvant Pathologic Characteristics Across Cohorts

Characteristic	Total (n = 243)	Training cohort (n = 146)	Internal validation cohort (n = 59)	External validation cohort (n = 38)	P

Neoadjuvant systemic therapy regimen, n (%)					0.290
Anthracycline- and taxane-based chemotherapy	114 (46.91)	72 (49.32)	24 (40.68)	18 (47.37)	
Dual HER2-targeted therapy combined with chemotherapy	101 (41.56)	61 (41.78)	25 (42.37)	15 (39.47)	
Single HER2-targeted therapy combined with chemotherapy	4 (1.65)	1 (0.68)	3 (5.08)	0 (0.00)	
Chemotherapy combined with immunotherapy	4 (1.65)	2 (1.37)	2 (3.39)	0 (0.00)	
Other chemotherapy regimens	20 (8.23)	10 (6.85)	5 (8.47)	5 (13.16)	
Lymphovascular invasion, n (%)					0.632
Absent	175 (72.02)	102 (69.86)	45 (76.27)	28 (73.68)	
Present	68 (27.98)	44 (30.14)	14 (23.73)	10 (26.32)	
ypT stage, n (%)					0.685
ypT0/is	85 (34.98)	48 (32.88)	23 (38.98)	14 (36.84)	
ypT1–4	158 (65.02)	98 (67.12)	36 (61.02)	24 (63.16)	
ypN stage, n (%)					0.682
ypN0	95 (39.09)	52 (35.62)	25 (42.37)	18 (47.37)	
ypN1	77 (31.69)	50 (34.25)	19 (32.20)	8 (21.05)	
ypN2	53 (21.81)	34 (23.29)	11 (18.64)	8 (21.05)	
ypN3	18 (7.41)	10 (6.85)	4 (6.78)	4 (10.53)	
Overall pathologic complete response, n (%)					0.809
Non-ypCR	172 (70.78)	104 (71.23)	40 (67.80)	28 (73.68)	
ypCR	71 (29.22)	42 (28.77)	19 (32.20)	10 (26.32)	

Note.—Unless otherwise indicated, data are numbers of patients, with percentages in parentheses. HER2 = human epidermal growth factor receptor 2; ypCR = pathologic complete response in the breast and regional lymph nodes (ypT0/is ypN0).

Table S7. Inter-reader agreement of structured MRI and Ultrasound imaging descriptors

Domain	Characteristic	κ (95% CI)	Percent agreement	N
MRI	BPE	0.896 (0.821–0.954)	0.956	205
	Depth localization	0.900 (0.846–0.947)	0.937	205
	Enhancement kinetics	0.890 (0.815–0.952)	0.956	205
	FGT	0.846 (0.762–0.917)	0.932	205
	Internal enhancement characteristics	0.756 (0.642–0.853)	0.917	205

	MF/MC	0.804 (0.721–0.882)	0.902	205
	Margin	0.808 (0.705–0.894)	0.932	205
	Morphology	0.785 (0.618–0.911)	0.956	205
	Peritumoral edema	0.842 (0.758–0.915)	0.927	205
	Shape	0.769 (0.662–0.865)	0.917	205
	T2 signal intensity	0.806 (0.677–0.912)	0.951	205
	Calcifications in a mass	0.787 (0.681–0.877)	0.922	205
	Echo pattern	0.816 (0.731–0.883)	0.907	205
**US**	Echogenic rind	0.797 (0.713–0.873)	0.898	205
**tumor**	Orientation	0.862 (0.767–0.941)	0.956	205
	Posterior features	0.817 (0.734–0.890)	0.907	205
	Shape	0.757 (0.656–0.847)	0.898	205
	Cortical thickening	0.807 (0.722–0.881)	0.907	205
**US**	Hilum	0.792 (0.695–0.873)	0.912	205
**ALN**	Margin	0.853 (0.780–0.920)	0.927	205
	Ratio of long/short	0.880 (0.810–0.940)	0.941	205

Supplementary Table S8. Distribution of Candidate Imaging Descriptors Between the Training and Internal Validation Cohorts

**Characteristic**	**Training cohort (n = 146)**	**Internal validation cohort (n = 59)**	***P***	**SMD**
MRI characteristics				
MF/MC, n (%)†			0.118	0.242
Absent	77 (52.74)	24 (40.68)		
Present	69 (47.26)	35 (59.32)		
Depth localization, n (%)†			<0.001	0.554
Anterior third	34 (23.29)	5 (8.47)		
Middle third	79 (54.11)	25 (42.37)		
Posterior third	33 (22.60)	29 (49.15)		
Morphology, n (%)			0.435	0.125
Mass	131 (89.73)	55 (93.22)		
NME	15 (10.27)	4 (6.78)		
FGT, n (%)			0.928	0.014
Fatty/Scattered	43 (29.45)	17 (28.81)		
Heterogeneous/Extremely dense	103 (70.55)	42 (71.19)		
BPE , n (%)			0.216	0.187
Minimal/Mild	109 (74.66)	39 (66.10)		
Moderate/Marked	37 (25.34)	20 (33.90)		
Shape, n (%)†			0.023	0.332
Oval/Lobulated/Round	22 (15.07)	17 (28.81)		
Irregular	124 (84.93)	42 (71.19)		

Margin, n (%)†			0.849	0.029
Circumscribed	28 (19.18)	12 (20.34)		
Indistinct/Spiculated	118 (80.82)	47 (79.66)		
Internal enhancement characteristics, n (%)			0.699	0.124
Homogeneous	7 (4.79)	3 (5.08)		
Heterogeneous	125 (85.62)	48 (81.36)		
Thick rim enhancement	14 (9.59)	8 (13.56)		
T2 signal intensity, n (%)†			0.024	0.324
Hyperintense	13 (8.90)	12 (20.34)		
Not hyperintense	133 (91.10)	47 (79.66)		
Peritumoral edema, n (%)†			0.51	0.102
Absent	49 (33.56)	17 (28.81)		
Present	97 (66.44)	42 (71.19)		
Enhancement kinetics, n (%)			0.714	0.056
Persistent/Plateau	110 (75.34)	43 (72.88)		
Washout	36 (24.66)	16 (27.12)		
US tumor characteristics				
Orientation, n (%)†			0.884	0.023
Parallel	120 (82.19)	49 (83.05)		
Non-parallel	26 (17.81)	10 (16.95)		
Shape, n (%)			0.827	0.034
Oval/Lobulated/Round	35 (23.97)	15 (25.42)		
Irregular	111 (76.03)	44 (74.58)		
Margin, n (%)			1.000	0.025
Circumscribed	9 (6.16)	4 (6.78)		
Indistinct/Angular/Microlobulated/Spiculated	137 (93.84)	55 (93.22)		
Echo pattern, n (%)			0.848	0.030
Hypoechoic	82 (56.16)	34 (57.63)		
Heterogeneous/Mixed solid and cystic	64 (43.84)	25 (42.37)		
Posterior features, n (%)			0.439	0.197
No posterior features	35 (23.97)	10 (16.95)		
Enhancement	18 (12.33)	6 (10.17)		
Shadowing	93 (63.70)	43 (72.88)		
Echogenic rind, n (%)†			0.584	0.085
Absent	68 (46.58)	25 (42.37)		
Present	78 (53.42)	34 (57.63)		
Calcifications in a mass, n (%)			0.396	0.128
Absent	27 (18.49)	14 (23.73)		
Present	119 (81.51)	45 (76.27)		
US axillary lymph-node characteristics				
Ratio of long/short , n (%)			0.347	0.144

<2	92 (63.01)	33 (55.93)		
≥2	54 (36.99)	26 (44.07)		
Margin, n (%)			0.956	0.009
Circumscribed	86 (58.90)	35 (59.32)		
Non-circumscribed	60 (41.10)	24 (40.68)		
Cortical thickening, n (%)			0.485	0.109
No/Uniform	94 (64.38)	41 (69.49)		
Non-uniform	52 (35.62)	18 (30.51)		
Hilum, n (%)†			0.732	0.053
Absent	108 (73.97)	45 (76.27)		
Present	38 (26.03)	14 (23.73)		

Note.—Unless otherwise indicated, data are numbers of patients, with percentages in parentheses. SMD = standardized mean difference; BPE = background parenchymal enhancement; FGT = fibroglandular tissue; LASSO = least absolute shrinkage and selection operator; MF/MC = multifocal and/or multicentric disease; US = ultrasound. † Descriptor retained by the training-cohort LASSO procedure.

Supplementary Table S9. Distribution of LASSO-Selected Imaging Descriptors Across Cohorts

Characteristic	Training cohort (n = 146)	Internal validation cohort (n = 59)	External validation cohort (n = 38)
MRI characteristics			
MF/MC, n (%)			
Absent	77 (52.74)	24 (40.68)	25 (65.79)
Present	69 (47.26)	35 (59.32)	13 (34.21)
Depth localization, n (%)			
Anterior third	34 (23.29)	5 (8.47)	7 (18.42)
Middle third	79 (54.11)	25 (42.37)	17 (44.74)
Posterior third	33 (22.60)	29 (49.15)	14 (36.84)
Margin, n (%)			
Circumscribed	28 (19.18)	12 (20.34)	13 (34.21)
Indistinct/spiculated	118 (80.82)	47 (79.66)	25 (65.79)
Shape, n (%)			
Oval/lobulated/round	22 (15.07)	17 (28.81)	14 (36.84)
Irregular	124 (84.93)	42 (71.19)	24 (63.16)
T2 signal intensity, n (%)			
Not hyperintense	133 (91.10)	47 (79.66)	26 (68.42)
Hyperintense	13 (8.90)	12 (20.34)	12 (31.58)
Peritumoral edema, n (%)			
Absent	49 (33.56)	17 (28.81)	18 (47.37)
Present	97 (66.44)	42 (71.19)	20 (52.63)
US tumor characteristics			

Orientation, n (%)			
Parallel	120 (82.19)	49 (83.05)	34 (89.47)
Non-parallel	26 (17.81)	10 (16.95)	4 (10.53)
Echogenic rind, n (%)			
Absent	68 (46.58)	25 (42.37)	24 (63.16)
Present	78 (53.42)	34 (57.63)	14 (36.84)
US axillary lymph-node characteristics			
Hilum, n (%)			
Absent	108 (73.97)	45 (76.27)	26 (68.42)
Present	38 (26.03)	14 (23.73)	12 (31.58)

Note.—Data are numbers of patients, with percentages in parentheses. LASSO = least absolute shrinkage and selection operator; MF/MC = multifocal and/or multicentric disease; US = ultrasound.

Supplementary Table S10. LASSO-Selected Imaging Descriptors and Corresponding Coefficients for Predicting ypN0

Modality/region	Descriptor	Comparison	LASSO coefficient	Direction for ypN0
MRI tumor (MT)	Peritumoral edema	Present vs absent	−0.820	Lower
	T2 Signal Intensity	Hyperintense vs not hyperintense	0.459	Higher
	Margin	Indistinct/spiculated vs circumscribed	−0.395	Lower
	Shape	Irregular vs oval/lobulated/round	−0.393	Lower
	Depth localization	Posterior third vs anterior third	−0.096	Lower
	MF/MC	Present vs absent	−0.091	Lower
Ultrasound tumor (UT)	Echogenic rind	Present vs absent	−0.524	Lower
Ultrasound tumor (UT)	Orientation	Non-parallel vs parallel	−0.231	Lower
Ultrasound axilla (UA)	Hilum	Present vs absent	0.735	Higher

Note.—Positive coefficients indicate a higher predicted probability of ypN0 and negative coefficients indicate a lower predicted probability, relative to the stated reference category. Coefficients are penalized LASSO coefficients and are not conventional logistic-regression odds ratios. LASSO = least absolute shrinkage and selection operator; MF/MC = multifocal and/or multicentric disease; MT = MRI tumor; UA = US axilla; US = ultrasound; UT = US tumor.

Supplementary Table S11. Associations between LASSO-Selected Imaging Descriptors and Clinicopathologic Variables

Imaging descriptor	Clinicopathologic variable	Cramer's V	P value	FDR-adjusted P value
**MT_Peritumoral edema**	Age	0.204	0.001	0.035
	Menopausal status	0.188	0.003	0.048
	cT stage	0.044	0.494	0.726
	cN stage	0.167	0.013	0.138
	Molecular subtype	0.131	0.124	0.472
	Ki-67 index	0.088	0.168	0.494
	Histologic type	0.031	0.776	0.873
	Histologic grade	0.046	0.546	0.763
**UA_Hilum**	Age	0.101	0.116	0.472
	Menopausal status	0.078	0.222	0.500
	cT stage	0.009	0.892	0.918
	cN stage	0.109	0.106	0.472
	Molecular subtype	0.141	0.090	0.472
	Ki-67 index	0.030	0.640	0.795
	Histologic type	0.093	0.207	0.497
	Histologic grade	0.063	0.402	0.674
**UT_Echogenic rind**	Age	0.107	0.095	0.472
	Menopausal status	0.082	0.203	0.497
	cT stage	0.031	0.625	0.795
	cN stage	0.095	0.157	0.494
	Molecular subtype	0.121	0.171	0.494
	Ki-67 index	0.038	0.551	0.763
	Histologic type	0.097	0.131	0.472
	Histologic grade	0.045	0.551	0.763
**MT_Margin**	Age	0.183	0.004	0.052
	Menopausal status	0.197	0.002	0.039
	cT stage	0.006	0.927	0.927
	cN stage	0.057	0.399	0.674
	Molecular subtype	0.177	0.022	0.198
	Ki-67 index	0.132	0.040	0.291
	Histologic type	0.088	0.314	0.622
	Histologic grade	0.074	0.326	0.622
**MT_T2 signal intensity**	Age	0.046	0.474	0.726
	Menopausal status	0.036	0.580	0.787
	cT stage	0.011	0.868	0.918
	cN stage	0.088	0.192	0.497
	Molecular subtype	0.047	0.821	0.896
	Ki-67 index	0.054	0.402	0.674
	Histologic type	0.056	0.701	0.809
	Histologic grade	0.067	0.373	0.674
**UT_Orientation**	Age	0.074	0.251	0.517
	Menopausal status	0.063	0.328	0.622
	cT stage	0.009	0.884	0.918

	cN stage	0.225	<0.001	0.030
	Molecular subtype	0.091	0.381	0.674
	Ki-67 index	0.007	0.908	0.920
	Histologic type	0.033	0.708	0.809
	Histologic grade	0.114	0.130	0.472
**MT_MF/MC**	Age	0.024	0.708	0.809
	Menopausal status	0.032	0.623	0.795
	cT stage	0.088	0.172	0.494
	cN stage	0.107	0.114	0.472
	Molecular subtype	0.112	0.220	0.500
	Ki-67 index	0.116	0.071	0.467
	Histologic type	0.009	0.886	0.918
	Histologic grade	0.062	0.414	0.677
**MT_Depth localization**	Age	0.076	0.493	0.726
	Menopausal status	0.078	0.474	0.726
	cT stage	0.173	0.026	0.211
	cN stage	0.065	0.629	0.795
	Molecular subtype	0.069	0.681	0.809
	Ki-67 index	0.044	0.791	0.876
	Histologic type	0.122	0.150	0.494
	Histologic grade	0.093	0.471	0.726
**MT_Shape**	Age	0.086	0.182	0.497
	Menopausal status	0.111	0.085	0.472
	cT stage	0.223	<0.001	0.030
	cN stage	0.081	0.231	0.503
	Molecular subtype	0.114	0.203	0.497
	Ki-67 index	0.027	0.669	0.809
	Histologic type	0.074	0.251	0.517
	Histologic grade	0.037	0.622	0.795

Note.—Cramer's V indicates association strength. P values were adjusted for multiple comparisons using the Benjamini-Hochberg false discovery rate (FDR) method. Age was dichotomized as >50 versus ≤50 years; cT stage as cT3–4 versus cT1–2; cN stage as cN2–3 versus cN1; Ki-67 index as ≥20% versus <20%; histologic type as IC-NST versus ILC/other; and histologic grade as grade I–II versus grade III. Histologic grade was available in 175 patients. HER2 = human epidermal growth factor receptor 2; HR = hormone receptor; IC-NST = invasive carcinoma of no special type; ILC = invasive lobular carcinoma; LASSO = least absolute shrinkage and selection operator; MT = MRI tumor; UA = US axilla; UT = US tumor.

Supplementary Table S12. Full Coefficient Specification of the Clinical and MT_UTA+Clinical Models

Model	Predictor / comparison	β coefficient	Standard error	OR (95% CI)	P
Clinical	Intercept	-1.221	0.362	0.29 (0.15–0.60)	<0.001
	cN2–3 vs cN1	-0.821	0.400	0.44 (0.20–0.96)	0.040
	HER2-positive vs HR+/HER2−	2.200	0.428	9.03 (3.90–20.90)	<0.001

	Triple-negative vs HR+/HER2−	0.543	0.668	1.72 (0.46–6.37)	0.417
MT_UTA+Clinical	Intercept	1.471	0.937	4.35 (0.69–27.30)	0.116
	cN2–3 vs cN1	-0.577	0.481	0.56 (0.22–1.44)	0.230
	HER2-positive vs HR+/HER2−	2.295	0.531	9.93 (3.51–28.08)	<0.001
	Triple-negative vs HR+/HER2−	-0.123	0.829	0.88 (0.17–4.49)	0.882
	MT_MF/MC: present vs absent	-0.749	0.521	0.47 (0.17–1.31)	0.151
	MT_Depth localization: middle third vs anterior third	-0.340	0.601	0.71 (0.22–2.31)	0.572
	MT_Depth localization: posterior third vs anterior third	-1.194	0.775	0.30 (0.07–1.38)	0.123
	MT_Margin: indistinct/spiculated vs circumscribed	-0.995	0.598	0.37 (0.11–1.20)	0.097
	MT_Shape: irregular vs oval/lobulated/round	-0.558	0.525	0.57 (0.20–1.60)	0.288
	MT_T2 signal intensity: hyperintense vs not hyperintense	1.755	0.933	5.78 (0.93–36.00)	0.060
	MT_Peritumoral edema: present vs absent	-1.379	0.518	0.25 (0.09–0.69)	0.008
	UT_Orientation: non-parallel vs parallel	-0.909	0.788	0.40 (0.09–1.89)	0.249
	UT_Echogenic rind: present vs absent	-1.005	0.510	0.37 (0.13–1.00)	0.049
	UA_Hilum: present vs absent	1.187	0.561	3.28 (1.09–9.84)	0.034

Note.—Coefficients were estimated in the training cohort with ypN0 as the outcome. Clinical includes cN stage and molecular subtype; MT_UTA+Clinical adds the nine locked LASSO-selected imaging descriptors. Reference categories were cN1, HR+/HER2−, MF/MC absent, anterior-third depth localization, circumscribed MRI margin, oval/lobulated/round MRI shape, non-hyperintense T2 signal, absent peritumoral edema, parallel US orientation, absent echogenic rind, and absent nodal hilum. CI = confidence interval; HR = hormone receptor; LASSO = least absolute shrinkage and selection operator; MT = MRI tumor; OR = odds ratio; SE = standard error; UA = US axilla; US = ultrasound; UT = US tumor.

Supplementary Table S13. Discrimination and Quantitative Calibration of the Clinical, MT_UTA, and MT_UTA+Clinical Models Across Cohorts

Cohort	Model	N	AUC (95% CI)	Brier	Calibration	Calibration

				score	intercept	slope
Training	Clinical	146	0.783 (0.707–0.859)	0.175	0.000	1.000
	MT_UTA	146	0.817 (0.741–0.892)	0.157	0.000	1.000
	MT_UTA+Clinical	146	0.886 (0.832–0.941)	0.128	0.000	1.000
Internal validation	Clinical	59	0.788 (0.672–0.904)	0.179	0.184	1.282
	MT_UTA	59	0.761 (0.638–0.884)	0.207	0.656	0.770
	MT_UTA+Clinical	59	0.858 (0.763–0.953)	0.161	0.597	0.848
External validation	Clinical	38	0.831 (0.697–0.964)	0.163	0.372	1.288
	MT_UTA	38	0.806 (0.667–0.944)	0.183	0.037	0.786
	MT_UTA+Clinical	38	0.931 (0.856–1.000)	0.104	-0.179	1.574

Note.—Lower Brier scores indicate better probabilistic accuracy; ideal calibration corresponds to an intercept of 0 and a slope of 1. Training-cohort calibration estimates represent apparent calibration. AUC = area under the receiver operating characteristic curve; CI = confidence interval; MT_UTA = MRI tumor + US tumor + US axilla descriptors.

Supplementary Table S14. Exploratory Subgroup Performance of the Imaging, Clinical, and Integrated Models in the Pooled Validation Cohort

Panel A. Model discrimination by molecular subtype

Model	HR+/HER2− (n = 41; ypN0/ypN1–3 = 5/36)	HER2-positive (n = 45; ypN0/ypN1–3 = 33/12)	Triple-negative (n = 11; ypN0/ypN1–3 = 5/6)
MT	0.908 (0.792–0.986)	0.727 (0.548–0.884)	0.700 (0.300–1.000)
UT	0.725 (0.530–0.875)	0.726 (0.561–0.874)	0.367 (0.067–0.667)
UA	0.844 (0.644–0.972)	0.572 (0.417–0.716)	0.517 (0.250–0.735)
MT_UT	0.917 (0.811–0.989)	0.788 (0.624–0.917)	0.733 (0.367–1.000)
MT_UA	0.958 (0.858–1.000)	0.739 (0.563–0.888)	0.667 (0.233–1.000)
UT_UA	0.906 (0.797–0.986)	0.679 (0.513–0.841)	0.367 (0.000–0.733)
MT_UTA	0.956 (0.867–1.000)	0.778 (0.609–0.909)	0.700 (0.300–1.000)
Clinical	0.594 (0.381–0.750)	0.591 (0.420–0.746)	0.617 (0.333–0.900)
MT_UTA+Clinical	0.928 (0.828–0.989)	0.804 (0.640–0.934)	0.700 (0.333–1.000)

Panel B. Descriptive incremental AUC by molecular subtype

Model comparison	HR+/HER2−	HER2-positive	Triple-negative
MT_UT vs MT	0.008	0.061	0.033

MT_UA vs MT	0.050	0.011	-0.033
MT_UTA vs MT	0.047	0.051	0.000
MT_UTA+Clinical vs MT_UTA	-0.028	0.027	0.000
MT_UTA+Clinical vs Clinical	0.333	0.213	0.083

Panel C. Performance of the MT_UTA+Clinical model by molecular subtype and cN stage

Subgroup variable	Subgroup	Total	ypN0	ypN1–3	AUC (95% CI)	SEN	SPE	PPV	NPV	ACC	ypN0 rate
Molecular subtype	HR+/HER2−	41	5	36	0.928 (0.828–0.989)	0.800	0.889	0.500	0.970	0.878	12.2%
Molecular subtype	HER2-positive	45	33	12	0.804 (0.640–0.934)	0.879	0.667	0.879	0.667	0.822	73.3%
Molecular subtype	Triple-negative	11	5	6	0.700 (0.333–1.000)	0.200	1.000	1.000	0.600	0.636	45.5%
cN stage	cN1	50	23	27	0.898 (0.807–0.968)	0.870	0.778	0.769	0.875	0.820	46.0%
cN stage	cN2–3	47	20	27	0.880 (0.767–0.967)	0.700	0.926	0.875	0.806	0.830	42.6%

Note.—The pooled validation cohort comprised the internal and external validation cohorts (n = 97). Subtype-specific analyses were exploratory and were performed without model refitting, feature reselection, or subgroup-specific cutoff optimization. AUCs were calculated from continuous predicted probabilities; 95% CIs were obtained using 2000 stratified bootstrap replicates. ΔAUC values in Panel B are descriptive absolute differences and were not subjected to formal between-model testing because of limited subgroup sample sizes. Sensitivity, specificity, PPV, NPV, and accuracy in Panel C were calculated using the training-cohort Youden cutoff (0.460361), applied unchanged. Subtype-specific NRI, IDI, calibration, and decision-curve analyses were not performed because of limited subgroup sample sizes, particularly in the triple-negative subgroup. ACC = accuracy; AUC = area under the receiver operating characteristic curve; cN = clinical nodal stage; HER2 = human epidermal growth factor receptor 2; HR = hormone receptor; IDI = integrated discrimination improvement; MT = MRI tumor; NPV = negative predictive value; NRI = net reclassification improvement; PPV = positive predictive value; SEN = sensitivity; SPE = specificity; UA = US axilla; UT = US tumor.

Supplementary Table S15 Exploratory Associations of Clinicopathologic Factors with ypCR and ypT0/is

Panel A. ypCR (71/243, 29.2%)

Variable / comparison	N	Univariable OR (95% CI)	P	Multivariable OR (95% CI)	P
Age, per 1-year increase	243	0.99 (0.96–1.02)	0.421	—	—
Postmenopausal vs premenopausal	243	1.07 (0.61–1.87)	0.819	—	—
cT3–4 vs cT1–2	243	0.37 (0.18–0.77)	0.008	0.42 (0.18–0.98)	0.044
cN2–3 vs cN1	243	0.68 (0.39–1.19)	0.175	—	—
Molecular subtype, overall†	243	—	<0.001	—	<0.001
HER2-positive vs HR+/HER2−	243	19.36 (8.20–45.70)	<0.00	16.95 (7.11–40.42)	<0.00

			1		1
Triple-negative vs HR+/HER2−	243	5.35 (1.69–16.92)	0.004	5.57 (1.72–18.00)	0.004
ER-positive vs ER-negative	243	0.29 (0.16–0.52)	<0.001	—	—
PR-positive vs PR-negative	243	0.30 (0.17–0.53)	<0.001	—	—
HER2-positive vs HER2-negative	243	11.49 (5.84–22.60)	<0.001	—	—
Ki-67 ≥20% vs <20%	243	3.49 (1.31–9.32)	0.012	2.19 (0.72–6.63)	0.165
ILC/other vs IC-NST	243	Not estimable‡	—	—	—
Grade III vs Grades I–II§	175	1.19 (0.57–2.46)	0.641	—	—

Panel B. ypT0/is (85/243, 35.0%)

Variable / comparison	N	Univariable OR (95% CI)	P	Multivariable OR (95% CI)	P
Age, per 1-year increase	243	0.99 (0.96–1.01)	0.280	—	—
Postmenopausal vs premenopausal	243	0.98 (0.57–1.67)	0.930	—	—
cT3–4 vs cT1–2	243	0.49 (0.26–0.94)	0.033	0.57 (0.27–1.21)	0.144
cN2–3 vs cN1	243	0.64 (0.38–1.09)	0.103	—	—
Molecular subtype, overall†	243	—	<0.001	—	<0.001
HER2-positive vs HR+/HER2−	243	13.73 (6.69–28.19)	<0.001	12.18 (5.88–25.24)	<0.001
Triple-negative vs HR+/HER2−	243	5.00 (1.87–13.37)	0.001	5.03 (1.85–13.70)	0.002
ER-positive vs ER-negative	243	0.21 (0.12–0.38)	<0.001	—	—
PR-positive vs PR-negative	243	0.23 (0.13–0.41)	<0.001	—	—
HER2-positive vs HER2-negative	243	8.74 (4.77–16.01)	<0.001	—	—
Ki-67 ≥20% vs <20%	243	3.06 (1.29–7.23)	0.011	1.94 (0.73–5.14)	0.183
ILC/other vs IC-NST	243	0.13 (0.02–1.03)	0.054	—	—
Grade III vs Grades I–II	175	1.34 (0.68–2.65)	0.403	—	—

Note.—Data are odds ratios (ORs), with 95% confidence intervals (CIs) in parentheses. For each endpoint, variables with P < 0.05 in univariable analysis were entered into the multivariable model. If molecular subtype and any of ER, PR, or HER2 status were simultaneously significant, only molecular subtype was entered because of conceptual overlap and collinearity. † For molecular subtype, the overall Wald P value is shown in the parent row; HR+/HER2− is the reference category. ‡ Complete separation precluded meaningful estimation for histologic type in the ypCR analysis. § Unknown histologic grades were excluded; Grades I–II were combined as the reference category. cN = clinical nodal stage; cT = clinical tumor stage; ER = estrogen receptor; HER2 = human epidermal growth factor receptor 2; HR = hormone receptor; IC-NST = invasive carcinoma of no special type; ILC = invasive lobular carcinoma; PR = progesterone receptor; ypCR = overall pathologic complete response.

Supplementary Table S16. Neoadjuvant Systemic Therapy Regimens by Molecular Subtype and Cohort

Molecular subtype	Regimen	Total, n (%)	Training, n (%)	Internal validation, n (%)	External validation, n (%)
HR+/HER2−	Anthracycline- and taxane-based chemotherapy	91 (79.8)	58 (79.5)	20 (87.0)	13 (72.2)
	Dual HER2-targeted therapy combined with chemotherapy	7 (6.1)	7 (9.6)	0 (0.0)	0 (0.0)
	Single HER2-targeted therapy combined with chemotherapy	0 (0.0)	0 (0.0)	0 (0.0)	0 (0.0)
	Chemotherapy combined with immunotherapy	0 (0.0)	0 (0.0)	0 (0.0)	0 (0.0)
	Other chemotherapy regimens	16 (14.0)	8 (11.0)	3 (13.0)	5 (27.8)
HER2-positive	Anthracycline- and taxane-based chemotherapy	5 (4.9)	3 (5.3)	0 (0.0)	2 (11.8)
	Dual HER2-targeted therapy combined with chemotherapy	92 (90.2)	53 (93.0)	24 (85.7)	15 (88.2)
	Single HER2-targeted therapy combined with chemotherapy	4 (3.9)	1 (1.8)	3 (10.7)	0 (0.0)
	Chemotherapy combined with immunotherapy	0 (0.0)	0 (0.0)	0 (0.0)	0 (0.0)
	Other chemotherapy regimens	1 (1.0)	0 (0.0)	1 (3.6)	0 (0.0)
Triple-negative	Anthracycline- and taxane-based chemotherapy	18 (66.7)	11 (68.8)	4 (50.0)	3 (100.0)

Dual HER2-targeted therapy combined with chemotherapy	2 (7.4)	1 (6.2)	1 (12.5)	0 (0.0)
Single HER2-targeted therapy combined with chemotherapy	0 (0.0)	0 (0.0)	0 (0.0)	0 (0.0)
Chemotherapy combined with immunotherapy	4 (14.8)	2 (12.5)	2 (25.0)	0 (0.0)
Other chemotherapy regimens	3 (11.1)	2 (12.5)	1 (12.5)	0 (0.0)

Note.—Data are numbers of patients, with percentages in parentheses. Percentages are calculated within each molecular subtype for the corresponding cohort. Regimen categories are defined in Supplementary Table S5, and standard regimen schedules, planned dose density, planned dose intensity, and cycle numbers are described in Supplementary Methods. HER2 = human epidermal growth factor receptor 2; HR = hormone receptor.

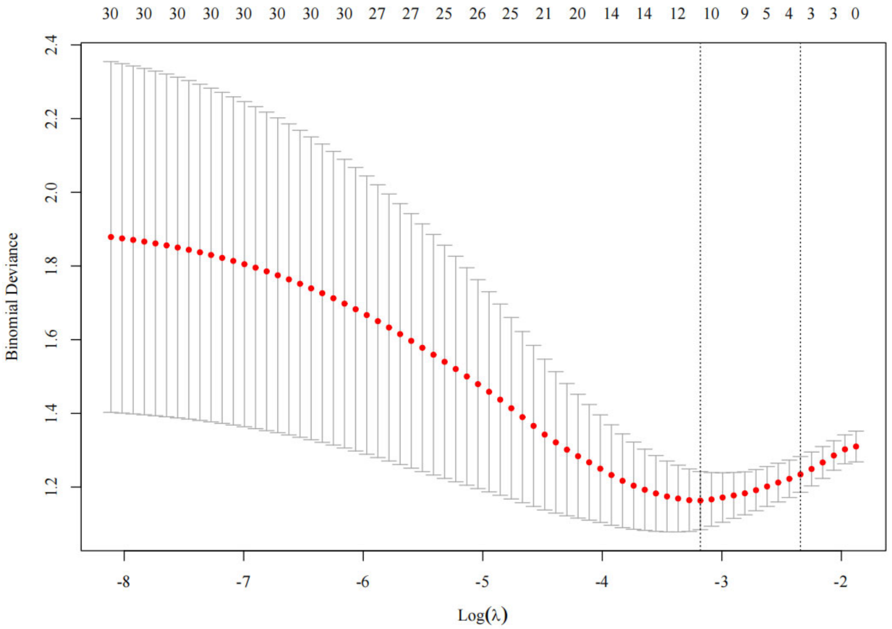

Figure S1. LASSO cross-validation error

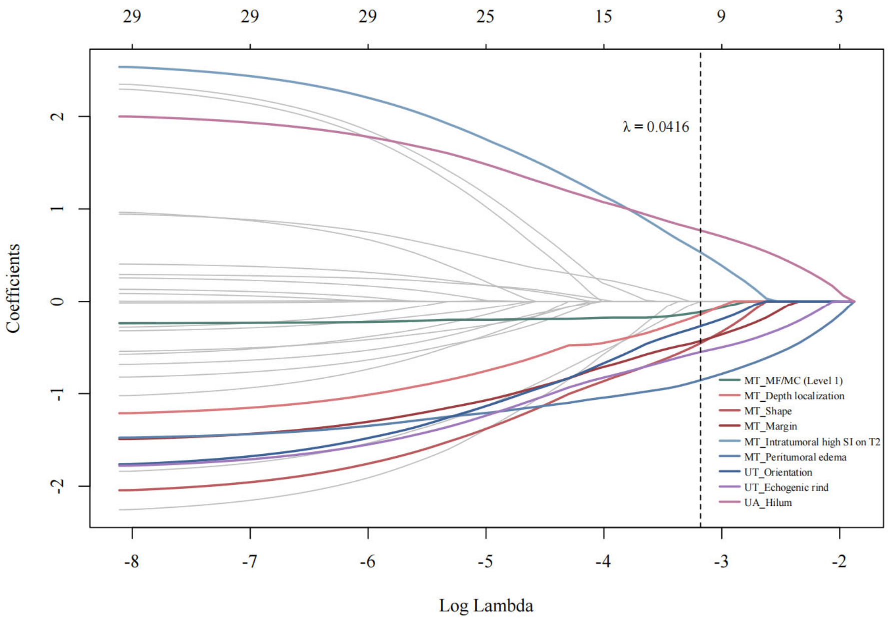

Figure S2. LASSO coefficient path

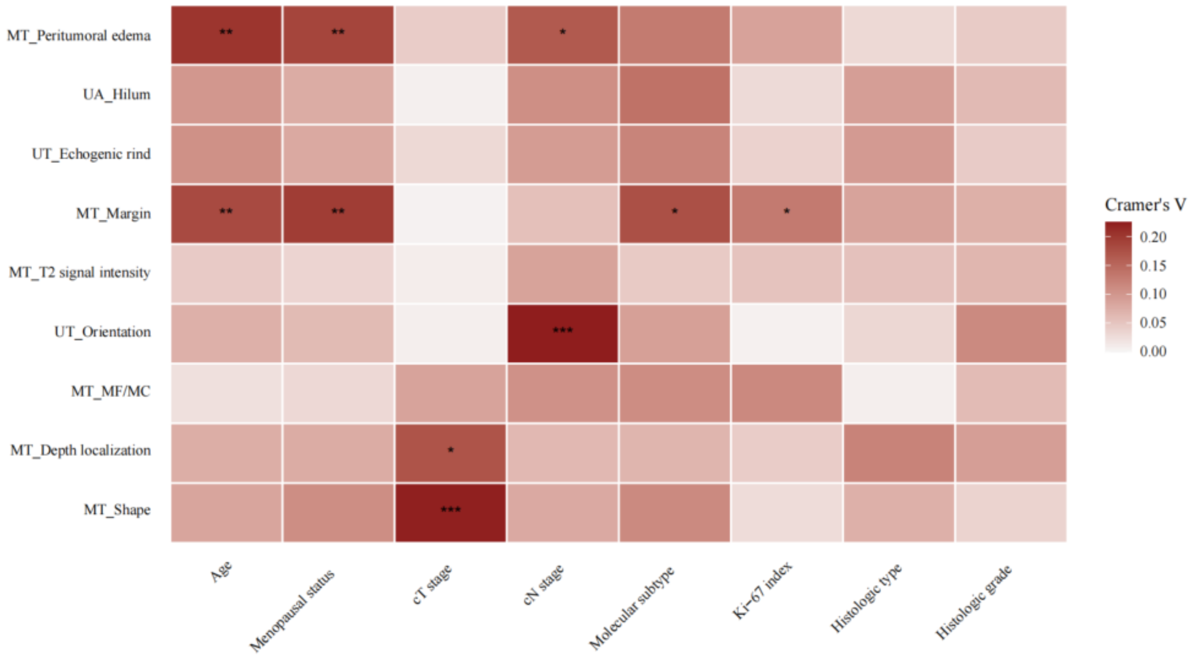

Figure S3. Association heatmap between LASSO-selected imaging descriptors and clinicopathologic variables. Color intensity indicates Cramer's V. Asterisks denote nominal significance before FDR correction (*P < .05, **P < .01, ***P < .001). Corresponding nominal and FDR-adjusted P values are provided in Supplementary Table S11. FDR = false discovery rate; LASSO = least absolute shrinkage and selection operator; MT = MRI tumor; UA = US axilla; UT = US tumor.

References

1. Morris EA, Comstock CE, Lee CH, et al. ACR BI-RADS® Magnetic Resonance Imaging. In: *ACR BI-RADS® Atlas, Breast Imaging Reporting and Data System*. Reston, VA: American College of Radiology, 2013
2. Newell MS, Destounis SV, Leung JWT, DeMartini WB, Lee CH, Eby PR. *ACR BI-RADS® v2025 Manual*. Reston, VA: American College of Radiology, 2025
3. Jirarayapong J, Portnow LH, Chikarmane SA, Lan Z, Gombos EC. High Peritumoral and Intratumoral T2 Signal Intensity in HER2-Positive Breast Cancers on Preneoadjuvant Breast MRI: Assessment of Associations With Histopathologic Characteristics. *American Journal of Roentgenology* 2024; 222:e2330280
4. Fischerova D, Garganese G, Reina H, et al. Terms, definitions and measurements to describe sonographic features of lymph nodes: consensus opinion from the Vulvar International Tumor Analysis (VITA) group. *Ultrasound in Obstet & Gyne* 2021; 57:861–879
5. Chung HL, Le-Petross HT, Leung JWT. Imaging Updates to Breast Cancer Lymph Node Management. *RadioGraphics* 2021; 41:1283–1299
6. Huang J-X, Liu F-T, Tan Y-T, et al. Enhancing detection of high-level axillary lymph node metastasis after neoadjuvant therapy in breast cancer patients with nodal involvement: a combined approach of axilla ultrasound and breast elastography. *Radiol med* 2024; 130:121–131
7. Hammond MEH, Hayes DF, Dowsett M, et al. American Society of Clinical Oncology/College of American Pathologists Guideline Recommendations for Immunohistochemical Testing of Estrogen and Progesterone Receptors in Breast Cancer. *JCO* 2010; 28:2784–2795
8. Wolff AC, Hammond MEH, Hicks DG, et al. Recommendations for Human Epidermal Growth Factor Receptor 2 Testing in Breast Cancer: American Society of Clinical Oncology/College of American Pathologists Clinical Practice Guideline Update. *JCO* 2013; 31:3997–4013
9. Roché H, Fumoleau P, Spielmann M, et al. Sequential Adjuvant Epirubicin-Based and Docetaxel Chemotherapy for Node-Positive Breast Cancer Patients: The FNCLCC PACS 01 Trial. *JCO* 2006; 24:5664–5671
10. Goldhirsch A, Winer EP, Coates AS, et al. Personalizing the treatment of women with early breast cancer: highlights of the St Gallen International Expert Consensus on the Primary Therapy of Early Breast Cancer 2013. *Annals of Oncology* 2013; 24:2206–2223
11. Asselain B, Barlow W, Bartlett J, et al. Long-term outcomes for neoadjuvant versus adjuvant chemotherapy in early breast cancer: meta-analysis of individual patient data from ten randomised trials. *The Lancet Oncology* 2018; 19:27–39
12. Gianni L, Pienkowski T, Im Y-H, et al. Efficacy and safety of neoadjuvant pertuzumab and trastuzumab in women with locally advanced, inflammatory, or early HER2-positive breast cancer (NeoSphere): a randomised multicentre, open-label, phase 2 trial. *The Lancet Oncology* 2012; 13:25–32
13. Untch M, Rezai M, Loibl S, et al. Neoadjuvant Treatment With Trastuzumab in HER2-Positive Breast Cancer: Results From the GeparQuattro Study. *JCO* 2010; 28:2024–2031
14. Schmid P, Cortes J, Dent R, et al. Overall Survival with Pembrolizumab in Early-Stage Triple-Negative Breast Cancer. *N Engl J Med* 2024; 391:1981–1991
15. Provenzano E, Bossuyt V, Viale G, et al. Standardization of pathologic evaluation and reporting of postneoadjuvant specimens in clinical trials of breast cancer: recommendations from an international working group. *Modern Pathology* 2015; 28:1185–1201
16. Amin MB, Greene FL, Edge SB, et al. The Eighth Edition AJCC Cancer Staging Manual: Continuing to build a bridge from a population-based to a more "personalized" approach to cancer staging. *CA A Cancer J Clinicians* 2017; 67:93–99
17. Cortazar P, Zhang L, Untch M, et al. Pathological complete response and long-term clinical benefit in

breast cancer: the CTNeoBC pooled analysis. *The Lancet* 2014; 384:164–172
